# *In vivo* cloning of up to 16 kb plasmids in *E*. *coli* is as simple as PCR

**DOI:** 10.1371/journal.pone.0183974

**Published:** 2017-08-24

**Authors:** Faqing Huang, Joseph Rankin Spangler, Allen Yang Huang

**Affiliations:** 1 Department of Chemistry and Biochemistry, University of Southern Mississippi, Hattiesburg, Mississippi, United States of America; 2 Oak Grove High School, Hattiesburg, Mississippi, United States of America; Imperial College London, UNITED KINGDOM

## Abstract

The precise assembly of defined DNA sequences into plasmids is an essential task in bioscience research. While a number of molecular cloning techniques have been developed, many methods require specialized expensive reagents or laborious experimental procedure. Not surprisingly, conventional cloning techniques based on restriction digestion and ligation are still commonly used in routine DNA cloning. Here, we describe a simple, fast, and economical cloning method based on RecA- and RecET-independent *in vivo* recombination of DNA fragments with overlapping ends using *E*. *coli*. All DNA fragments were prepared by a 2-consecutive PCR procedure with Q5 DNA polymerase and used directly for transformation resulting in 95% cloning accuracy and zero background from parental template plasmids. Quantitative relationships were established between cloning efficiency and three factors–the length of overlapping nucleotides, the number of DNA fragments, and the size of target plasmids–which can provide general guidance for selecting *in vivo* cloning parameters. The method may be used to accurately assemble up to 5 DNA fragments with 25 nt overlapping ends into relatively small plasmids, and 3 DNA fragments into plasmids up to 16 kb in size. The whole cloning procedure may be completed within 2 days by a researcher with little training in cloning. The combination of high accuracy and zero background eliminates the need for screening a large number of colonies. The method requires no enzymes other than Q5 DNA polymerase, has no sequence restriction, is highly reliable, and represents one of the simplest, fastest, and cheapest cloning techniques available. Our method is particularly suitable for common cloning tasks in the lab where the primary goal is to quickly generate a plasmid with a pre-defined sequence at low costs.

## Introduction

The advancement of recombinant DNA techniques has dramatically expanded our ability to manipulate DNA for the purpose of gene alteration, fusion protein construction, DNA and protein library generation, metabolic pathway assembly, and synthetic chromosome and genome construction [1–4]. Conventional cloning techniques [5, 6] rely on restriction enzyme digestion and inefficient DNA ligation, require multiple laborious steps of DNA manipulation involving enzymatic reactions and DNA purification, and have relatively low cloning efficiencies. The requirement for restriction sites puts a severe burden on the DNA sequence, particularly for relatively large plasmids where unique restriction sites at desired locations are difficult to find, and often leaves behind “scars” in the sequence. Furthermore, restriction digestion can rarely be completed 100% even with extended incubation time and thus leaves behind residual template plasmids that contribute to significant backgrounds. To overcome these limitations, various sequence- and ligation-independent cloning methods have been developed. These methods, such as LIC-PCR [7], SLIC [8], In-fusion [9], USER [10], and Gibson Assembly [11], require overlapping ends of DNA fragments and are proceeded by the creation of overhangs through either 3’→5’ exonuclease activity or deoxyuridine excision, followed by complementary strand annealing and DNA gap-filling by a DNA polymerase. Additionally, DNA may be joined together by a DNA ligase. While these methods are efficient in assembling multiple DNA fragments into seamless circular DNA molecules before transformation, they require expensive reagents and additional steps following DNA fragment preparation. Alternatively, phage-based recombination systems (e.g., GATEWAY) [12], overlap extension PCR (OEC) [13, 14], and *in vitro* recombination methods (SLiCE) [15] have been developed to assemble DNA fragments into plasmids.

An *in vivo* recombination principle was first demonstrated in *E*. *coli* with DNA fragments possessing homologous sequences more than 3 decades ago [16], revealing the high dependency of recombination efficiency on the length of overlapping DNA sequences. Since then, cloning based on *in vivo* recombination has been accomplished in yeast [17–20] and *E*. *coli* [21, 22]. Although *E*. *coli* strains with Red/RecET recombinases are good hosts for *in vivo* cloning [23–25], derivatives of the commonly used DH5α lab strain without recA and Red/RecET activities have been used successfully for *in vivo* assembly of plasmids [21, 26–30]. Unlike conventional cloning and SLIC methods, *in vivo* recombination cloning requires only DNA fragments with overlapping ends, which can be prepared by PCR to eliminate the need for additional enzymes such as exonuclease and DNA ligase for DNA manipulation.

Notwithstanding its simplicity, the *in vivo* recombination principle has not been widely adopted for general cloning purposes. Its lack of broad acceptance may be attributed to its relatively low efficiency, little understanding of quantitative correlations among cloning efficiency, the length of overlapping DNA ends, the number of DNA fragments, and the size of plasmids. In addition, the relatively low recombination efficiency makes it critical to eliminate template DNA plasmids by DpnI treatment following PCR. Otherwise, the template DNA plasmids may contribute to significantly high backgrounds. However, the simplicity of *in vivo* cloning principle is starting to draw increasing attention of researchers as reflected by the recent surge in reports that focus on improving the practical aspects of *in vivo* cloning to make it simple, fast, and efficient compared with other cloning techniques. These studies included varying lengths of overlapping nucleotides from 16 to 50 nt, different numbers of separately prepared DNA fragments, and plasmids of various sizes [26–28, 30]. Interestingly, a recent report described a simultaneous preparation of multiple DNA fragments with overlapping ends in a single-tube PCR reaction [29]. All mentioned methods used DpnI digestion of PCR products to remove template plasmids before transformation to reduce colony background.

DH5α and DH10β derivatives (derived from *E*. *coli* K12 strain) are commonly used competent cells for cloning. To ensure stable DNA amplification, the activity of nonspecific endonuclease I (*endA1*) is eliminated and DNA recombination activity (*recA1*) is reduced in these cells. The recombination-impaired *E*. *coli* strains nevertheless retains sufficient recombinase activity for *in vivo* assembly of DNA fragments with homologous ends [21, 26–30]. Although the exact mechanism remains unknown [28], the non-conventional *recA*-independent recombination activity requires homologous DNA sequences and is enhanced by the absence of the recA protein and exonucleases [31, 32]. Cell extracts of these recA-negative strains can be used for *in vitro* recombination of DNA fragments with homologous ends before transformation into competent cells (SLiCE) [15], indicating that commonly used recA-negative E. coli cells contain all the necessary enzymatic activities for homologous DNA recombination. However, these activities do not comprise the cells’ ability for stable DNA propagation, as the evidence from this study will show.

We hereby present a simple, fast, and efficient method for accurately assembling multiple DNA fragments into plasmids at a minimum cost based on the homologous recombination capabilities of the common DH5α strain of *E*. *coli*. Extensive recombination experiments were conducted to derive quantitative relationships between cloning efficiency and three factors–the length of overlapping nucleotides, the number of DNA fragments, and the size of plasmids. These relationships reveal intrinsic virtues and limitations of *in vivo* cloning in *E*. *coli*, and serve as valuable practical guides for setting appropriate experimental parameters to achieve the desired cloning outcomes. Our *in vivo* cloning method may be performed by a researcher with little training in cloning to construct plasmids up to 16 kb within 2 days. For larger plasmids, the method may be used to quickly assemble PCR DNA pieces into intermediary plasmids, which can then be used as building blocks for constructing target plasmids by *in vitro* SLIC methods such as Gibson Assembly [11].

All DNA fragments used for assembly were prepared from low concentrations of template plasmids (≤ 10 fg/μL) by a 2-consecutive PCR procedure and used directly for transformation into chemically competent *E*. *coli* cells, leading to 95% positive colonies, while no colonies (zero background) were observed from the same concentrations of template plasmids alone. The combination of high cloning accuracy and zero background of this method made it unnecessary to screen for positive colonies, in contrast to other cloning methods that normally screen a large number of transformants. The success of the method itself merely depends on the preparation of high quality DNA fragments with homologous ends for recombination, and therefore the cost of cloning can be reduced to that of primers and commonly used reagents for transformation and plasmid preparation after the initial investment in a high-fidelity DNA polymerase. Both high fidelity with 3’→5’ exonuclease activity and high processivity are integral in maintaining DNA quality prepared by PCR. In this regard, Q5 DNA polymerase (NEB) is unmatched by other DNA polymerases [33], and works well for the purpose of preparing high quality DNA fragments for *in vivo* cloning through homologous recombination.

## Materials and methods

### Materials

Taq DNA polymerase, Q5 Hot Start DNA polymerase, dNTP, restriction enzymes, DNA markers, High Efficiency NEB 5-alpha chemically competent cells, and pUC19 were purchased from New England Biolabs (NEB). DNA oligonucleotides (S1–S4 Tables) were synthesized by either Integrated DNA Technologies or Life Technologies. pGLO was from Bio-Rad. pETite N-His Kan and pETite N-His SUMO were purchased from Lucigen Technologies. pcDNA3.1/Hygro (+) and pEGFP-N1 were from Invitrogen and Clontech, respectively. pET28a-Ec.coaA [34] was a gift from Erick Strauss (Addgene plasmid # 50386). pET28a-Ec.coaD (pESC106) [35] was a gift from Tadhg Begley & Erick Strauss (Addgene plasmid # 50388). pIk and pk-Dcr were kindly provided by the Ballard lab [36] and the Cullen lab [37], respectively.

### Generation of DNA fragments by PCR

Primer pairs for generating DNA fragments with varying overlapping ends by PCR were designed using the freely accessible SnapGene Viewer program. First, a target plasmid sequence was compiled in a MS word document. DNA sequence segments were copied from template plasmids and joined together. Changes to the sequence such as adding a peptide linker coding sequence were made to yield the final plasmid sequence, which was copied to make a SnapGene file of the target plasmid. From the program, DNA fragments from different parent plasmids were located. A pair of forward and reverse primers were designed at each joint so that they had the desired number of overlapping nucleotides (OL). The OL region could reside completely on the either side of the junction or astride the joint symmetrically or asymmetrically. Although it is a common practice that PCR primer pair should have similar annealing temperatures (Ta), we found that high quality DNA bands could be obtained by PCR with primer pairs of quite different Ta’s, as long as the annealing temperature was set according to the lower Ta. High GC content at the 3’ ends of primers should be avoided, due to their potential to cause low yields and non-specific DNA bands.

DNA fragments for the construction of all the plasmids in this study were prepared by PCR reactions using template plasmids and Q5 DNA polymerase according to the manufacturer’s protocol. In a typical PCR experiment, the total reaction volume was kept low to save costs. A typical 6–10 μL PCR reaction was sufficient for DNA quality analysis by gel electrophoresis and transformation. To avoid background colony formation from template plasmids, 2 consecutive PCR reactions were performed for each DNA fragment so that the final concentration of the template plasmids was diluted below 10 fg/μL. As an example, the first step PCR of 8 μL reaction contained 0.4 μL of 1 ng/μL template plasmid, leading to 50 pg/μL template plasmid concentration (20-fold dilution). The PCR sample was diluted 250 fold and then used as the template for the second PCR procefure following the same protocol. The final template plasmid concentration was 10 fg/μL. The extension time at 72°C is determined by the size of DNA fragment, 30 s/kB. The appropriate PCR cycle number is both size- and, to a lesser extent, sequence-dependent. As a general rule of thumb, use 16/20/25/30 cycles for 2/4/8/12 kb DNA fragments. If the primer annealing sequences are GC-rich, such as the 5’ end of EGFP, the PCR efficiency is normally lower. Attempts to amplify target DNA fragments directly from 10 fg/μL template plasmids were not successful.

Agarose gel electrophoresis was used to analyze DNA fragments from all PCR reactions to ensure good quality of expected DNA bands in terms of the correct size, intensity, and clean PCR reaction. An aliquot of 0.5–1.5 μL was loaded, along with a known quantity of DNA markers, onto a 0.8–1.5% agarose gel containing EtBr. After 30–60 min running at 2–3 W (80–100 V), the gel was scanned by a phosphorimager (BioRad) under the Cy3 fluorescence setting and quantified by Quantity One (BioRad).

### Construction of plasmids by *in vivo* recombination of DNA fragments with overlapping ends

All the plasmids in this study were assembled *by E*. *coli in vivo* assembly of 2–5 DNA fragments with perfectly matched overlapping ends. After analysis by gel electrophoresis, DNA fragments from PCR reactions were used directly without either DpnI digestion or purification for transformation into high efficiency competent *E*. *coli* DH5α cells (transformation efficiency of ~1 x 10^9^ cfu/μg pUC19, but the actual transformation efficiency under our conditions was more than 10 times lower). Instead of the typical DNA transformation using 50 μL or more competent cells, we opted to use only 10 μL of aliquoted cells to save costs. DNA fragments (0.6 μL from each of PCR reactions) were mixed prior to transformation. One microliter of the DNA mixture was added to and gently mixed with 10 μL competent cells, and the cells were incubated for 30 min on ice. Following the standard 30 s heat shock at 42°C on a metal thermal block and 2 min incubation on ice, 120 μL of SOC medium was added to each transformation. After vigorously shaking the cells for 1 h at 37°C, all the cells from each transformation were plated onto a single petri dish, and incubated for 20 h at 37°C.

Background colonies from contamination could be a serious issue. It is imperative to use filter pipette tips and frequently change gloves to minimize the risk of contamination.

### Construction of template plasmids pcDNA3 Kan and pDSA used in multiple DNA fragment assembly

pcDNA3 Kan (S1 Sequence, 5437 bp) was constructed from pcDNA3.1/Hygro (+) by replacing AmpR with KanR through 2-fragment assembly with 18-nt overlapping ends. Fragment 1 (F1) (4452 bp) was generated from the template plasmid pcDNA3.1/Hygro (+) by PCR using the primer pair atatctagagaattcgtcGAAGAATCTGCTTAGGGTTAGG/ctcgatgagtttttctaaCTGTCAGACCAAGTTTACTCAT (annealing sequences to the template are indicated by Uppercase letters and the lowercase letters are homologous sequences with the ends of the other DNA fragment). F2 (1003 bp) was amplified from pETite N-His Kan using the primer pair TTAGAAAAACTCATCGAGC/GACGAATTCTCTAGATATCG. One of the isolated plasmid (without sequencing) was used as the template for the construction of plasmids pIKB, pDcEG, pDSADE in this study.

Similarly, pDSA (S2 Sequence, 7037 bp) was assembled by 3 DNA fragments with 25-nt homologous ends. The 3 DNA fragments–F1 (5798 bp), F2 (976 bp), and F3 (341 bp)–were generated by PCR from the template plasmids pET28a-Ec.coaD, pET28a-Ec.coaA, and pETite N-His SUMO, respectively, using the primer pairs TAACTCCGTCGACAAGC/CGCTAACTTCGCCAT, ATGAGTATAAAAGAGCAAACG/tgcggccgcaagcttgtcgacggagttaTTTGCGTAGTCTGACCT, and tcaggcgctgatggcgaagttagcgCAGGACTCAGAAGTC/ttaacgtttgctcttttatactcatACCTCCAATCTGTTC. One of the isolated plasmid was used directly as the template for the construction of pDSADE.

### Colony analysis

Developed colonies from each transformation were photographed and the number of colonies were counted manually on a computer screen. For GFP-containing plasmids, bright fluorescent colonies could be distinguished easily from non-fluorescent ones. For other plasmids, colony PCR of randomly selected colonies were performed to assess cloning accuracy. A stock solution was made to contain 1X buffer, 2.5 mM MgCl_2_, 0.25 mM dNTPs, 0.05 U/μL *Taq* DNA polymerase, and 0.2 μM primers. Cells from colonies were hand-picked up by pipette tips and released to the PCR tube by rubbing the tip against the bottom plastic wall. Fifteen microliters of the PCR stock solution was added to each tube, followed by heating 95°C for 10 minutes and 30 standard PCR cycles. Agarose gel electrophoresis was then used to analyze the result. PCR primers were designed to reside within 2 separate DNA fragments so that a positive PCR band of expected size reflected the correct joining of 2 DNA fragments. For effective colony PCR and subsequent analysis by agarose gel, PCR product sizes of around 200 bp were found to be optimal.

### Sequence analysis

Plasmids from randomly selected colonies of each construct were isolated. Restriction digestion followed by agarose gel electrophoresis was used to confirm cloning accuracy. Additionally, 2 isolated plasmids from each construct were sequenced at joint regions by the Sanger method. Finally, whole plasmid sequencing by Next Generation Sequencing (NGS) was conducted at Massachusetts General Hospital’s DNA core facility (https://dnacore.mgh.harvard.edu/new-cgi-bin/) on two plasmids–one each from randomly selected 2.6 kb pGFP and 16.1 kb pDSADE.

## Results and discussion

### Cloning strategy

The basic principle of *E*. *coli in vivo* recombination was demonstrated in 1985 [16]. However, its application in cloning has been limited by a lack of understanding of the factors affecting recombination events in *E*. *coli*. The goal of this study was to develop a simple, cost effective, and efficient cloning method for broad applications based on *E*. *coli in vivo* recombination. To achieve the goal, experiments were designed to investigate how 3 factors–the length of overlapping nucleotides, the number of overlapping DNA fragments, and the size of target plasmids–correlate with *E*. *coli* recombination events. Four plasmid constructs of different sizes–pGFP, pIkB, pDcEG, and pDSADE with respective 2.6, 6.3, 11.9, and 16.1 kb–were assembled by *in vivo* recombination of overlapping DNA fragments under varying conditions.

**Fig 1:**
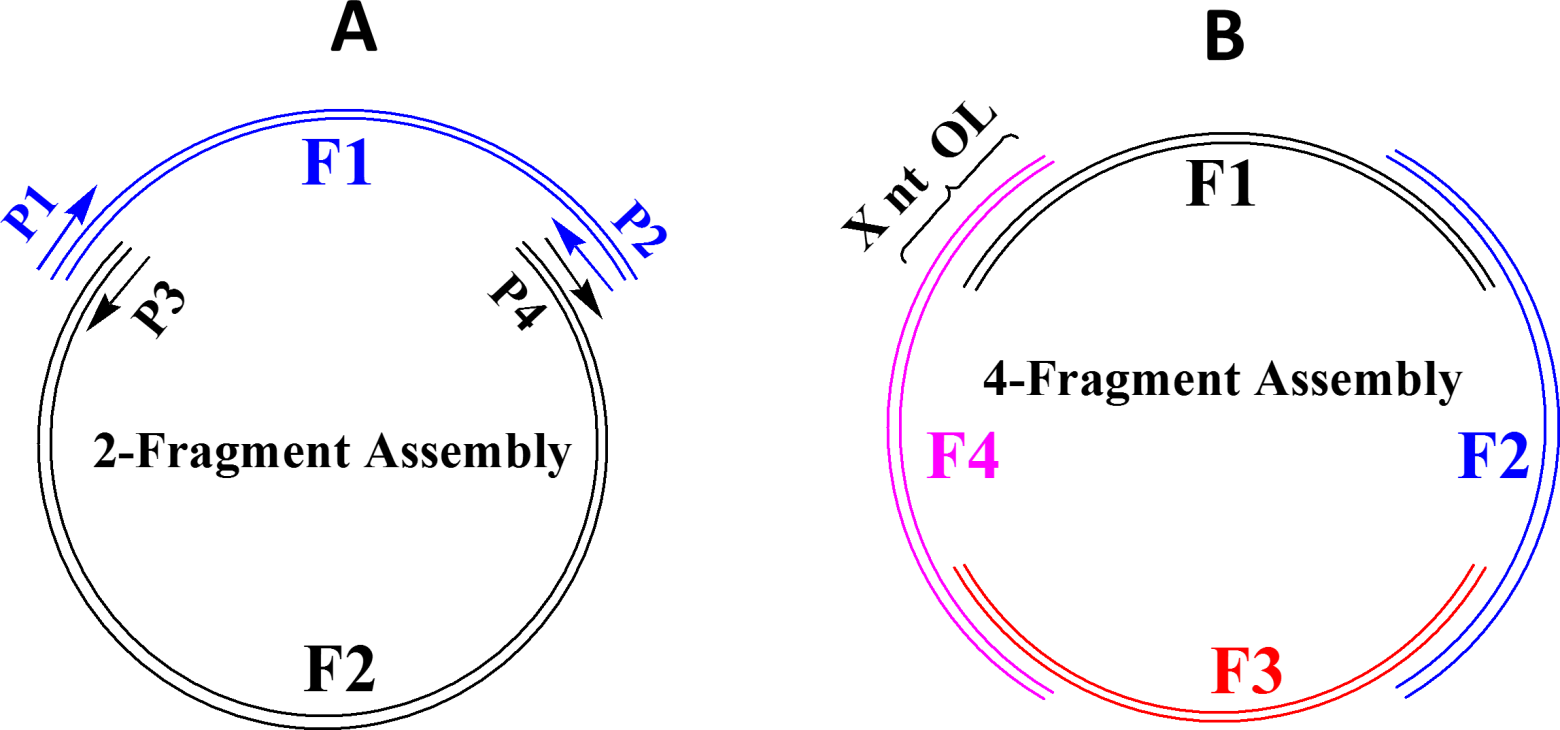
Plasmid assembly by *E*. *coli in vivo* recombination of multiple DNA fragments (F1, F2, F3, and F4) with overlapping ends. (**A**) Assembly by 2 DNA fragments, (**B**) Assembly by 4 DNA fragments. The length of overlapping ends is indicated by X nt OL (e.g. 18 nt OL). The DNA fragments should contain a replication origin (Ori), an antibiotic resistant gene (AmpR or KanR), a promoter, and one or more target genes (or gene fragments). Each of the DNA fragments is generated by PCR from DNA templates and a pair of primers (P1/P2, P3/P4) with high fidelity Q5 DNA polymerase.

The simple requirement for *E*. *coli in vivo* recombination is the perfectly matched overlapping nucleotides between DNA fragments. Fig 1 illustrates the *in vivo* assembly scheme to construct a plasmid by multiple DNA fragments with overlapping ends. Although DNA fragments may be prepared by different methods such as chemical synthesis, gene synthesis, PCR, or restriction digestion of DNA, we opted to synthesize all the DNA fragments individually by PCR using the high fidelity Q5 DNA polymerase and to use them directly without further treatment. Compared with other DNA polymerases such as *Taq*, Q5 not only has a low error rate of nucleotide incorporation but also often yields clean and bright DNA bands, particularly for relatively large DNA fragments (6–12 kb).

**Fig 2:**
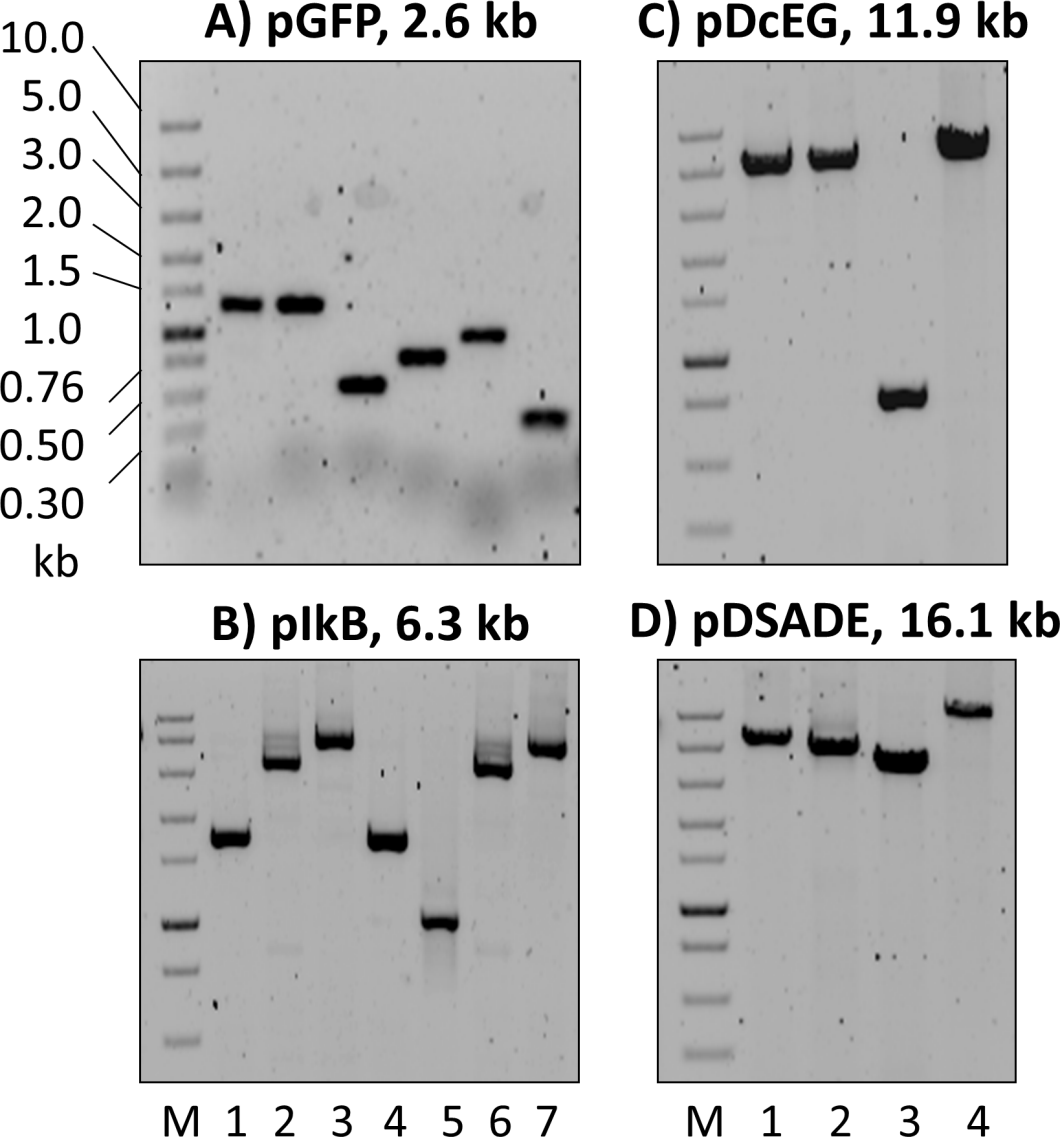
DNA fragments of varying sizes (from 344–11,792 bp) prepared by PCR used for the construction of plasmids. (**A**) pGFP (2.6 kb), (**B**) pIkB (6.3 kb), (**C**) pDcEG (11.9 kb), and (**D**) pDSADE (16.1 kb). The 1 kb band in the DNA marker lane (M, 1.5 μL NEB Fast DNA ladder) contained 8.1 ng DNA. Aliquots of 1.5 μL PCR reactions were loaded onto 0.8–1.5% agarose gels containing EtBr. After running 45–60 min at 80–100 V, the gels were scanned by a phosphorimager under Cy3 fluorescence setting. The sequences of constructed plasmids, PCR primers, and overlapping regions are included in S1–S4 Tables and S1–S6 Sequences.

For all cloning strategies, it is imperative to limit the undesired colony background below a defined level. Tests under our transformation conditions with 10 fg plasmids of different sizes showed zero background. Therefore, to avoid cloning background from parental plasmids used as templates for PCR, 2 consecutive PCR reactions were performed so that the concentration of the original template plasmid could reach below 10 fg/μL. Representative PCR reactions for the construction of pGFP, pIkB, pDcEG, and pDSADE are shown in Fig 2.

While restriction digestion of parental plasmids by DpnI following PCR is a common strategy to reduce the background in cloning [27–29, 38], DpnI does not have optimal activity in PCR buffers and cannot fully eradicate parental plasmids even with lengthy incubation time. As a result, background colonies resulting from template plasmids can be reduced but may not be eliminated by DpnI digestion of PCR products. On the other hand, our procedure of 2 consecutive PCR amplification reactions could easily bring the concentration of template plasmids below a certain level so that no background colonies were formed. The resulting zero background is a significant advantage over other cloning techniques, especially when the number of colonies is low, such as the *in vivo* assembly of multiple DNA fragments in this study.

To analyze PCR products, phosphorimager scanning was more sensitive than commonly used Gel Doc systems, allowing DNA detection and analysis from a small volume of the PCR reaction (e.g. 0.5 μL). A drawback was that this high sensitivity could also detect small particles (invisible to human eyes) either inside the gel, on the gel surface or on the tray surface below the gel. However, the particle-generated dots in the image could be easily distinguished from DNA bands, thereby rarely affecting DNA detection and quantitation (Fig 2).

A PCR reaction was considered successful if it produced a bright DNA band of the expected size, was free of other bands, and yielded 15–40 ng/μL DNA (irrespective of the DNA size). Theoretically, each PCR cycle should increase the amount of target DNA by a factor of 2 before reaching saturation. However, the actual PCR efficiency would usually be lower. We found that a factor of 1.7 could be used to successfully predict the cycle number of most PCR reactions. For example, if a DNA fragment from a successful PCR reaction was diluted 5000 times and used as the template for the next PCR reaction, 16 PCR cycles would normally produce the target DNA with similar concentration as the first PCR reaction (1.7^16^ = 4866). Occasionally, a weaker-than-expected DNA band was observed, but 2–5 more PCR cycles were often sufficient to bring the target DNA concentration to the normal range. When the DNA size increased, we observed lower amplification efficiency. Additional 4/9/14 cycles were often needed to reach saturation for 4/8/12 kb DNA fragments. Furthermore, the final target DNA concentration tended to be lower than that of < 7 kb DNA fragments. Finally, when the primer annealing sequences are GC-rich (e.g., the 5’ end of EGFP), the PCR efficiency tends to be lower.

While Garcia-Nafria et al. [29] recently reported the generation of multiple DNA fragments with overlapping ends by PCR in a single-tube reaction, we were unsuccessful in multiple attempts to amplify 3 DNA fragments with homologous ends by a single PCR reaction (using the same primers to amplify the same DNA fragments in Fig 2C & 2D). Even with a single DNA fragment preparation, we often had to adjust PCR conditions such as Ta and cycle number to obtain high quality PCR products. In general, PCR efficiency goes down with the increasing DNA size as we described previously [39]. As a result, larger DNA fragments tend to be amplified less efficiently than smaller ones under the same PCR conditions in a single-tube reaction setting. We have observed that even similar DNA sizes may have different PCR amplification efficiencies. Furthermore, synthesizing DNA fragments with overlapping ends requires primers with partial complimentary sequences, which may complicate the primer annealing efficiency. Taken together, our experience suggests that PCR amplification of multiple DNA fragments with overlapping ends in a single-tube reaction as reported by Garcia-Nafria et al. [29] may be difficult to accomplish in routine experiments. Preparation of individual DNA fragments by separate PCR reactions as described in this study is therefore highly recommended.

### Length of overlapping nucleotides-recombination efficiency relationship from 2-fragment assembly to construct 2.6 kb pGFP

A simple and reliable method was highly desirable for screening a large number of colonies from multiple transformation experiments under different conditions. The bright cycle 3 GFP [40] was selected as the visual indicator for successful plasmid assembly by recombination. Four DNA fragments with overlapping ends–Lac promoter from pUC19, cycle 3 GFP from pGLO, Ori and KanR from pETite N-His Kan–were prepared by PCR with 18 nt OL ends (S1 Table). Direct transformation of a mixture of the 4 DNA fragments into competent DH5α cells resulted in 0–4 bright green fluorescent colonies after 20 h incubation at 37°C on LB media supplemented with Kanamycin. IPTG was not needed for the induction of GFP expression under the control of the Lac promoter. However, bright fluorescence took time to develop, requiring 16–20 h at 37°C and longer time at 35°C. The plasmid pGFP (S3 Sequence) was purified from one of the colonies and used as a template for further generation of DNA fragments by PCR.

**Fig 3:**
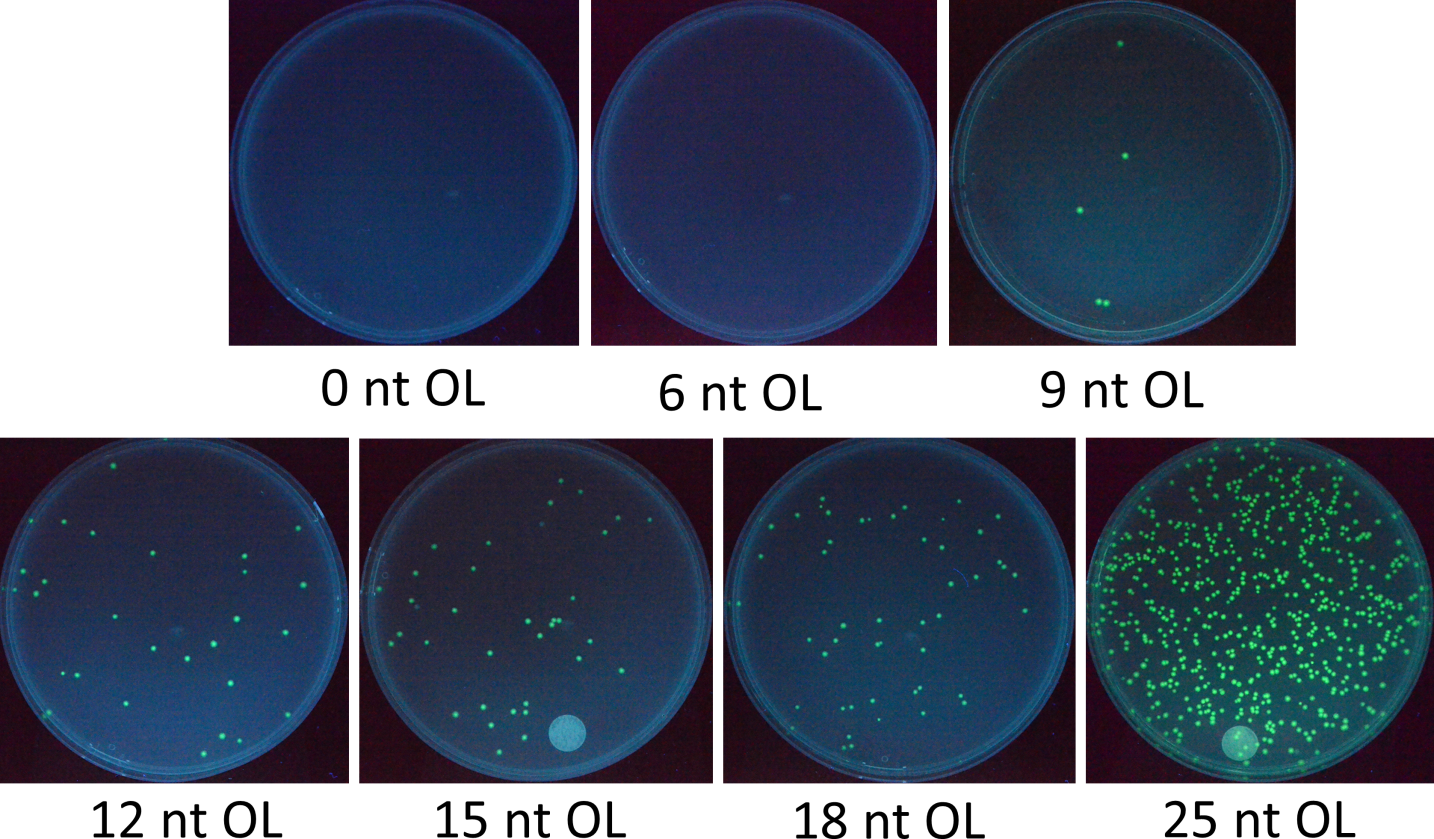
Colonies from 2-fragment assembly of varying lengths of overlapping nucleotides (0–25 nt OL) to construct the pGFP plasmid. The zero background can be seen from 0 and 6 nt OL. Increasing the OL size generates a higher number of colonies.

**Fig 4:**
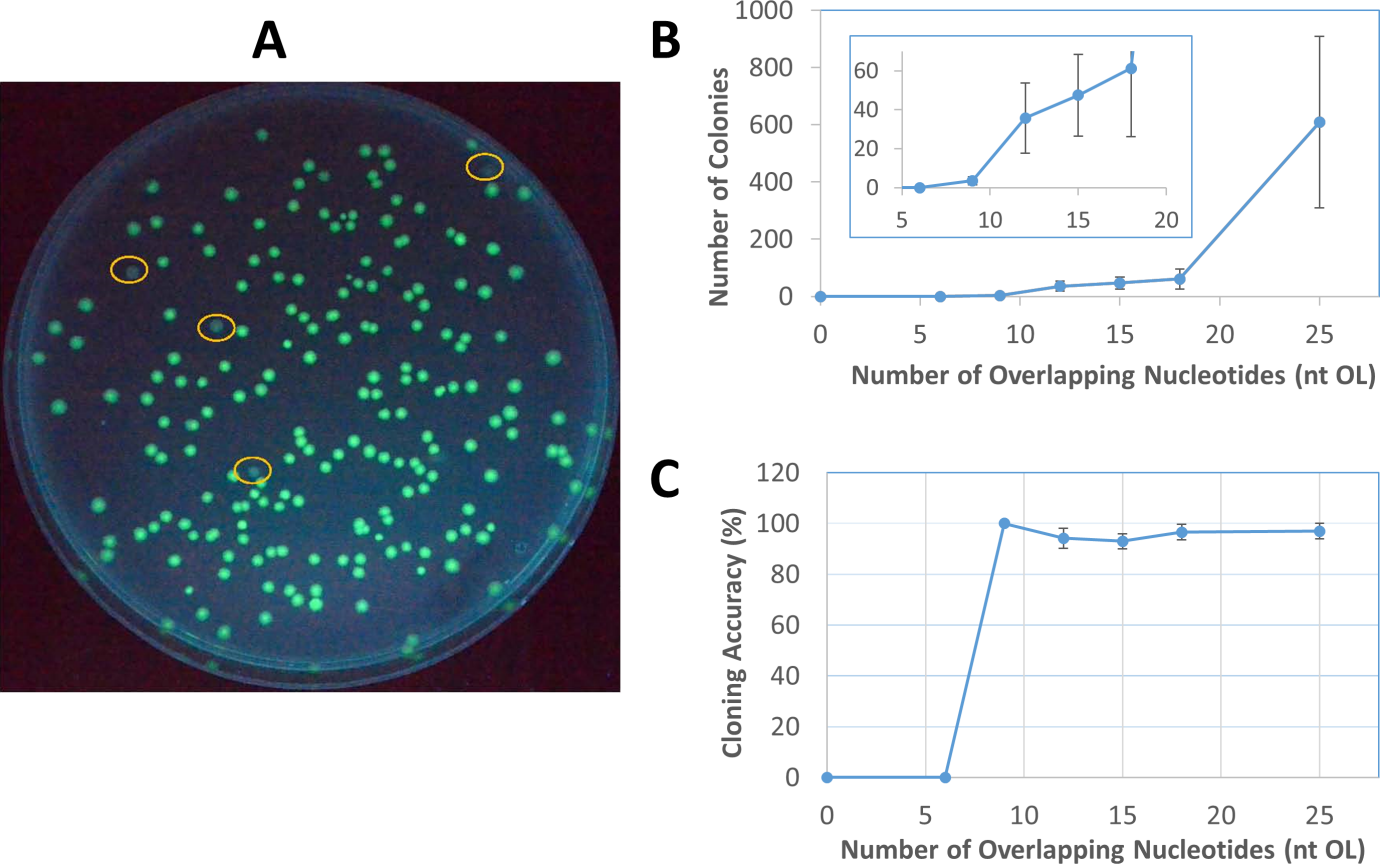
The relationship between cloning efficiency and the length of overlapping ends. (**A**) Identification of positive and negative colonies by fluorescence. The circled colonies were non-fluorescent, which were used to calculate cloning accuracy of plasmid assembly by *E*. *coli in vivo* recombination. (**B**) The relationship between the number of colonies and overlapping nucleotides through 2-fragment assembly to construct pGFP. The data were from 2–6 independent experiments for each point, and the error bar represents the range of variation. (**C**) High cloning accuracy of pGFP 2-fragment assembly, regardless of the length of overlapping nucleotides. The 9 nt OL point had apparent 100% accuracy and no variation from 2 independent experiments, but it only had 7 colonies in total.

The first set of experiments was designed to assess cloning efficiency of *in vivo* recombination from 2 DNA fragments with varying lengths of overlapping ends–from 0–25 nt OL. The 2 fragments split GFP in the middle and were each about half the size of the plasmid pGFP, (S1 Table). Using this system, only successful recombinations maintaining the GFP reading frame would produce fluorescence and therefore represent the seamless assembly of DNA fragments. In this study, cloning efficiency is defined by 2 separate parameters–the number of total colonies from a competent cell transformation and cloning accuracy as determined by the percentage of positive green fluorescent colonies. As shown in Fig 3, transformation of 2 DNA fragments with 6 nt OL or less did not yield a single colony, indicating that 6-nt DNA homologous sequences were too short for *in vivo* recombination. At the same time, the 0 and 6 nt OL plates demonstrated the zero background from the parental plasmid template pGFP used for PCR to generate the DNA fragments. However, starting from 9 nt OL, the number of positive colonies rose steadily then sharply from 18 nt OL to 25 nt OL. The relationship between the colony number and overlapping nucleotides for this relatively small 2.6 kb plasmid pGFP, derived from 2–6 independent transformation experiments at each point, is shown in Fig 4B. While the variation of colony number is relatively large (typically about 50% of the average), Fig 4B indicates a strong correlation between the colony number and the length of overlapping nucleotides. From 18 nt OL to 25 nt OL, the number of colonies increases by about an order of magnitude.

Positive and negative colonies could be easily identified visually, as shown in Fig 4A. Cloning accuracy from all transformation experiments on pGFP assembly by recombination stayed unchanged at about 95%, irrespective of the length of overlapping nucleotides (Fig 4C). Recombination from 2 fragments with 9 nt OL yielded only a few colonies, but all were fluorescent (a total of 7 positive colonies) in 2 independent transformations, giving the apparent “100%” cloning accuracy without variation. However, if more independent transformation experiments had been performed to yield a higher number of total colonies, the cloning accuracy might be expected to approach 95% based on the relative constant cloning accuracy of 2-fragment recombination from different lengths of overlapping nucleotides. From Fig 4B & 4C, while the number of total colonies strongly correlated with the lengths of overlapping nucleotides of the 2 DNA fragments, the cloning accuracy remained almost constant at 95%.

According to Watt et al. [16], *in vivo* recombination events in *E*. *coli* increases exponentially with OL from 20 to 74 nt then rises linearly from 74 to 313 nt. Therefore, *in vivo* assembly of DNA fragments with long OL tends to yield a relatively high number of colonies. However, long OL places a significant burden on DNA fragment preparation. Such preparation requires long DNA primers for PCR, which not only increases the cost of the primer but also gives rise to the potential for mispriming and undesired DNA fragments. Previous studies on *in vivo* DNA assembly typically used 30–50 nt OL [27, 28, 30] to produce a reasonable number of colonies. In this study, we sought to limit OL at 25 nt so that the optimal balance among the primer cost, mispriming, and *in vivo* assembly efficiency could be found. Most of the primers could be designed at ≤ 40 nt to take the advantage of the Invitrogen Value Oligos offering at the same low price. From Fig 4B & 4C, the significant increase in colony number from 18 to 25 nt OL suggests that 25 nt OL should be used for *in vivo* assembly if a large number of colonies is desired. On the other hand, shorter OL down to 9 nt may be sufficient to yield a lower number of correctly assembled plasmids.

Garcia-Nafria et al. [29] investigated how the length (nt OL) under a constant Tm and binding strength under a constant length of homologous regions affected *in vivo* assembly efficiency. Although it was found that Tm correlated better with cloning efficiency, we chose to use the length of homologous regions without the constraint of Tm for simplicity. As seen in Fig 4B, without the constant Tm constraint used by Garcia-Nafria et al. [29], the length of homologous regions correlated well with the number of colonies. It is worth noting that our colony numbers were generally lower than many reported studies based on the same *in vivo* recombination principle [14, 27, 28, 30] for three reasons to save costs–using shorter homologous regions (≤ 25 nt), a smaller volume of competent cells (10 μL), and a smaller amount of DNA fragments (total of 1 μL PCR DNA, 15–40 ng) for transformation.

### Cloning efficiency from multiple DNA fragment assembly to construct pGFP

**Fig 5:**
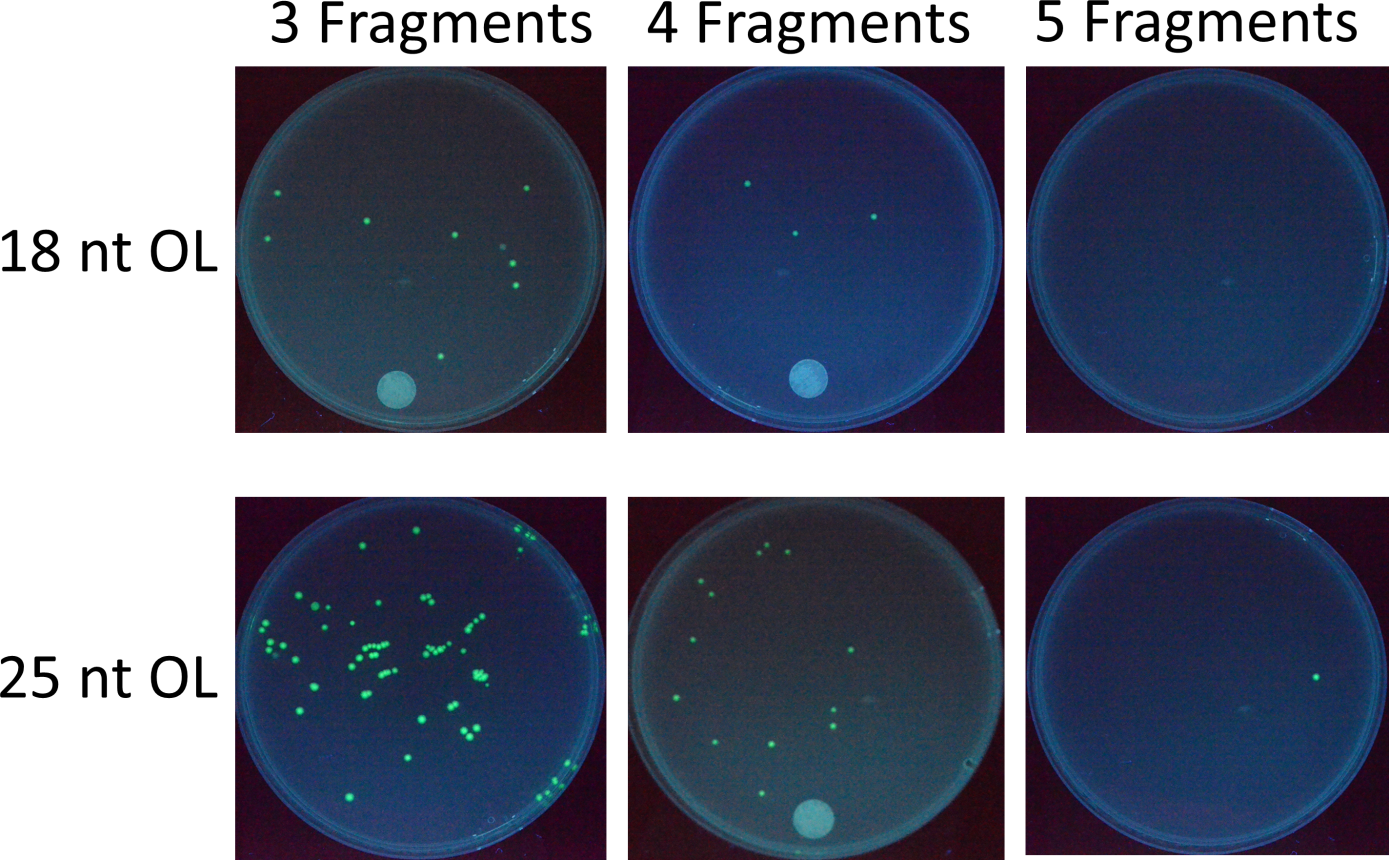
Colonies from *in vivo* assembly of 3–5 overlapping DNA fragments with 18 nt OL and 25 nt OL to construct pGFP. While increasing the OL size produces more colonies, the number of colonies decreases with increasing number of DNA fragments.

**Fig 6:**
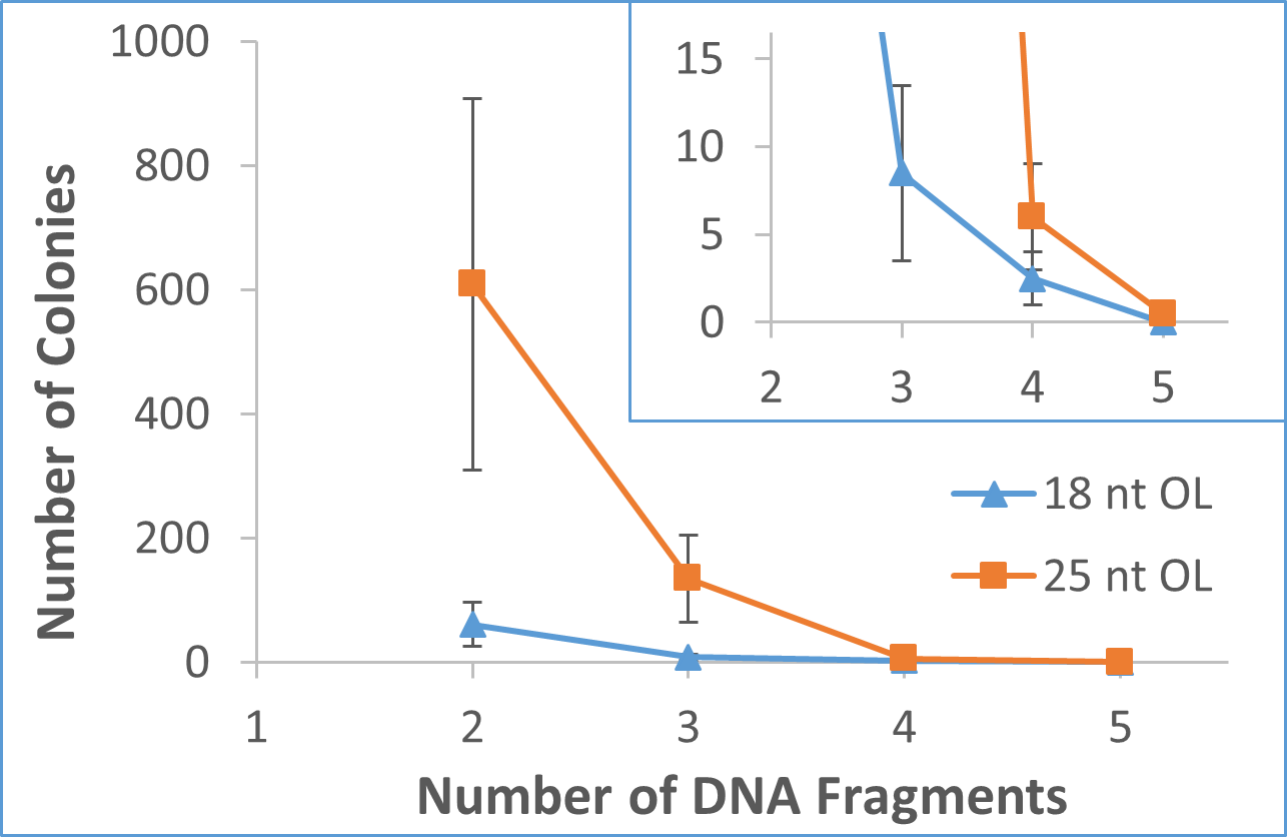
The correlation between the number of colonies and the number of DNA fragments (2–5) with 18 nt OL and 25 nt OL to construct pGFP. For both 18 and 25 nt OL assemblies, the colony number rapidly decreases with the number of DNA fragments. The error bar represents the range of variation.

Following the extensive investigation on 2-fragment recombination to construct pGFP (Figs 3 and 4), multiple fragment assembly experiments using 18 nt OL and 25 nt OL were conducted to construct the same pGFP (S1 Table). Fig 5 shows the results from a typical set of transformations. Combined with the results from 2-fragment assembly (Fig 4B), the number of colonies decreased rapidly with the number of DNA fragments for both 18 nt OL and 25 nt OL, as demonstrated in Fig 6. For each additional DNA fragment, the colony number fell approximately by an order of magnitude. For 18 nt OL, 4-fragment assembly yielded around 2 positive colonies, while no colony was obtained from 5-fragment recombination. On the other hand, 4-fragment assembly of 25 nt OL produced around 6 positive colonies, and two independent 5-fragment recombination experiments produced 0 & 1 fluorescent colony. For all the transformations from multiple DNA fragment assembly, cloning accuracy remained the same around 95%.

### Construction of 6.3 kb pIkB, 11.9 kb pDcEG, and 16.1 kb pDSADE from multiple DNA fragment assembly

pGFP is a relatively small plasmid (2.6 kb). In order to expand the applicability of *in vivo* recombination principle to plasmid cloning in general, three larger plasmids pIkB (6.3 kb, S4 Sequence), pDcEG (11.9 kb, S5 Sequence), and pDSADE (16.1 kb, S6 Sequence) were constructed by *in vivo* assembly of DNA fragments with homologous ends. For the construction of pIkB, assembly of 2 DNA fragments prepared by PCR using the backbone vector pcDNA3 Kan (S1 Sequence) and pIk yielded pIkB-containing colonies. One purified plasmid from the colonies was used as the template to prepared DNA fragments with 18 nt OL or 25 nt OL to study 2- and 3-fragment assembly by recombination (S2 Table). For the construction of pDcEG, 3 DNA fragments with either 18 nt OL or 25 nt OL were prepared by PCR using pcDNA3 Kan, pk-Dcr (human dicer) [37], and pEGFP-N1 as the templates. One isolated plasmid from the 25 nt OL assembly was used as the template for 2-fragment assembly experiments with 18 nt OL and 25 nt OL (S3 Table). Finally, for the construction of pDSADE, DNA fragments of 25 nt OL were generated using pDcEG and pDSA (S2 Sequence) as the templates (S4 Table).

**Fig 7:**
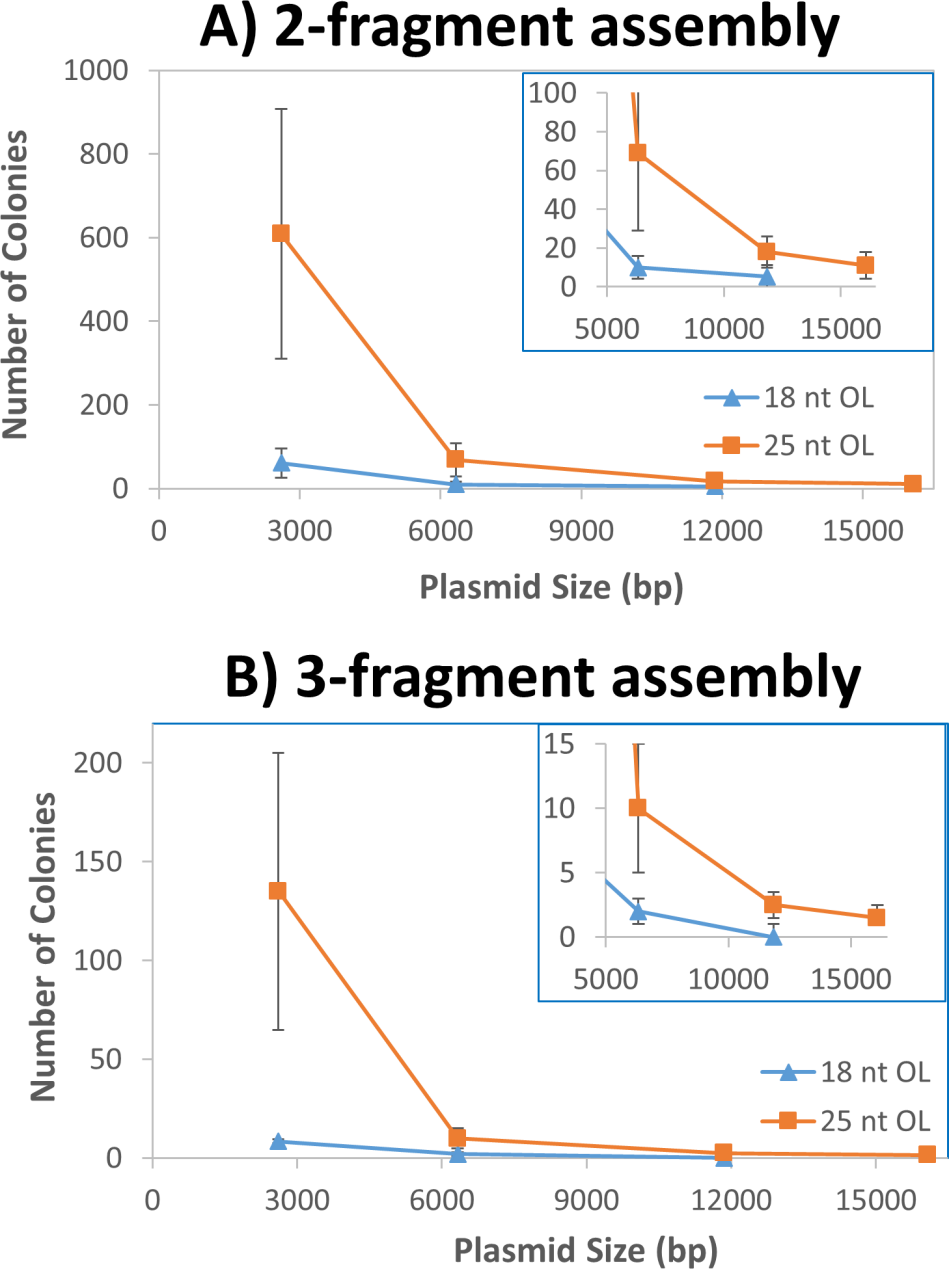
Correlation of plasmid size and the number of colonies. (A) The relationship between the number of colonies and plasmid size through 2-fragment assembly with 18 nt OL and 25 nt OL. (B) The relationship between the number of colonies and plasmid size from 3-fragment assembly with 18 nt OL and 25 nt OL. The error bar represents the range of variation.

Combined with the results from pGFP, Fig 7 shows that the number of colonies decreased steadily with the increase of the plasmid size for both 2- and 3-fragment assemblies of either 18 nt OL or 25 nt OL. From 2.6 kb to 6.3 kb, the number of colonies fell approximately by an order of magnitude for both 2- and 3-fragment assemblies. The number dropped further about 3-fold when the plasmid size increased from 6.3 kb to 11.8 kb. Although the 3-fragment assembly with 18 nt OL into pDcEG generated only 0–2 colonies, all colonies contained desired plasmids, due to the zero background. From the figure, 12 kb plasmid represented a size limit for *in vivo* assembly of 3-fragments with 18 nt OL. As a result, *in vivo* assembly of 16.1 kb pDSADE was studied with only 25 nt OL DNA fragments. When the plasmid increased from 11.9 kb to 16.1 kb, the plasmid number decreased 30–70% for 2- and 3-fragment assemblies. While the colony number was low, the majority of colonies were expected to contain correct sequences.

**Fig 8:**
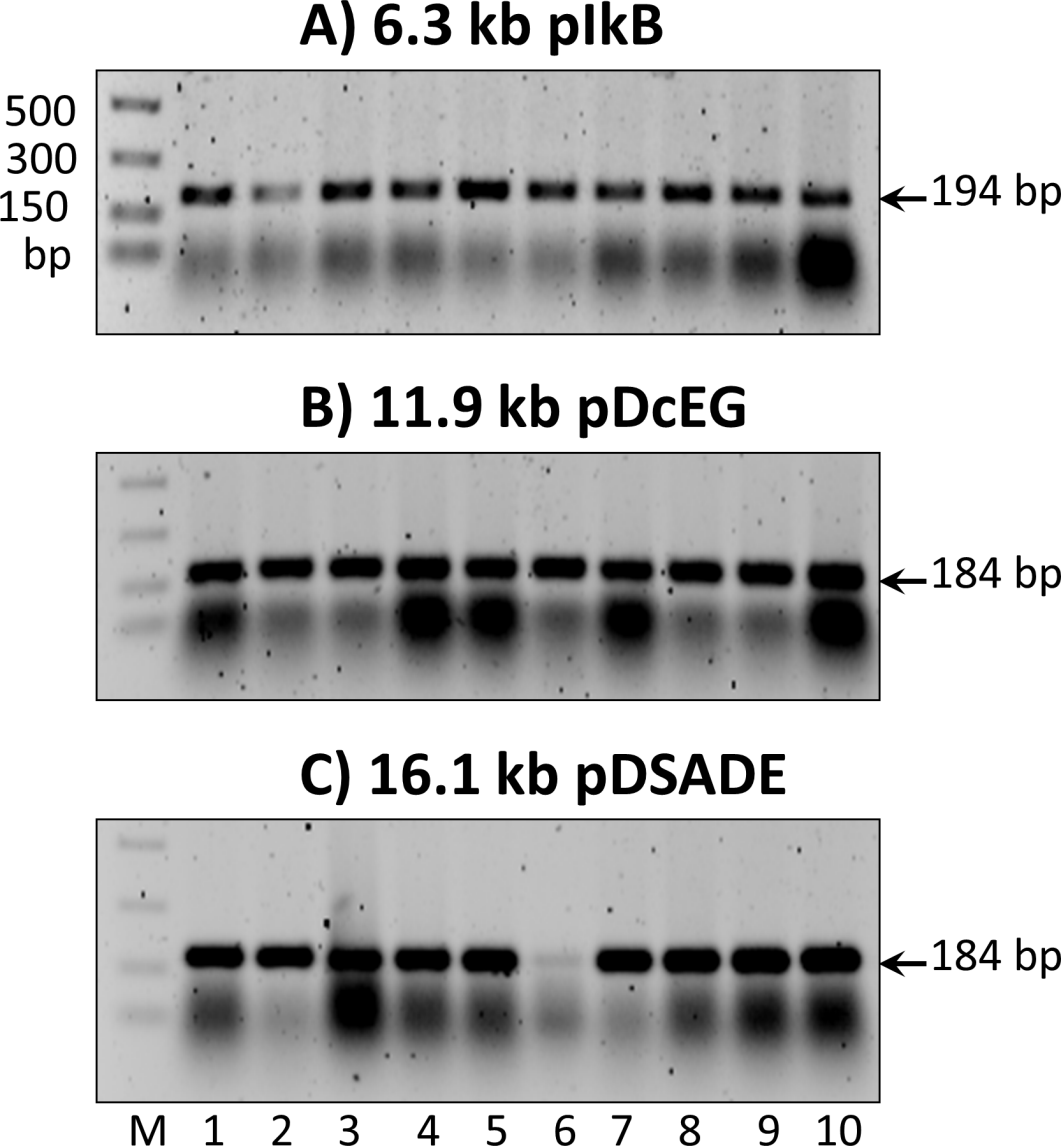
**Identification of positive colonies by colony PCR from the construction of (A) pIkB, (B) pDcEG, and (C) pDSADE.** The expected correct DNA products are marked on the right sides of the gels. The smear bands below the correct DNA product bands are not consistent from different PCR reactions, but their identity and reasons for inconsistency are unknown.

**Fig 9:**
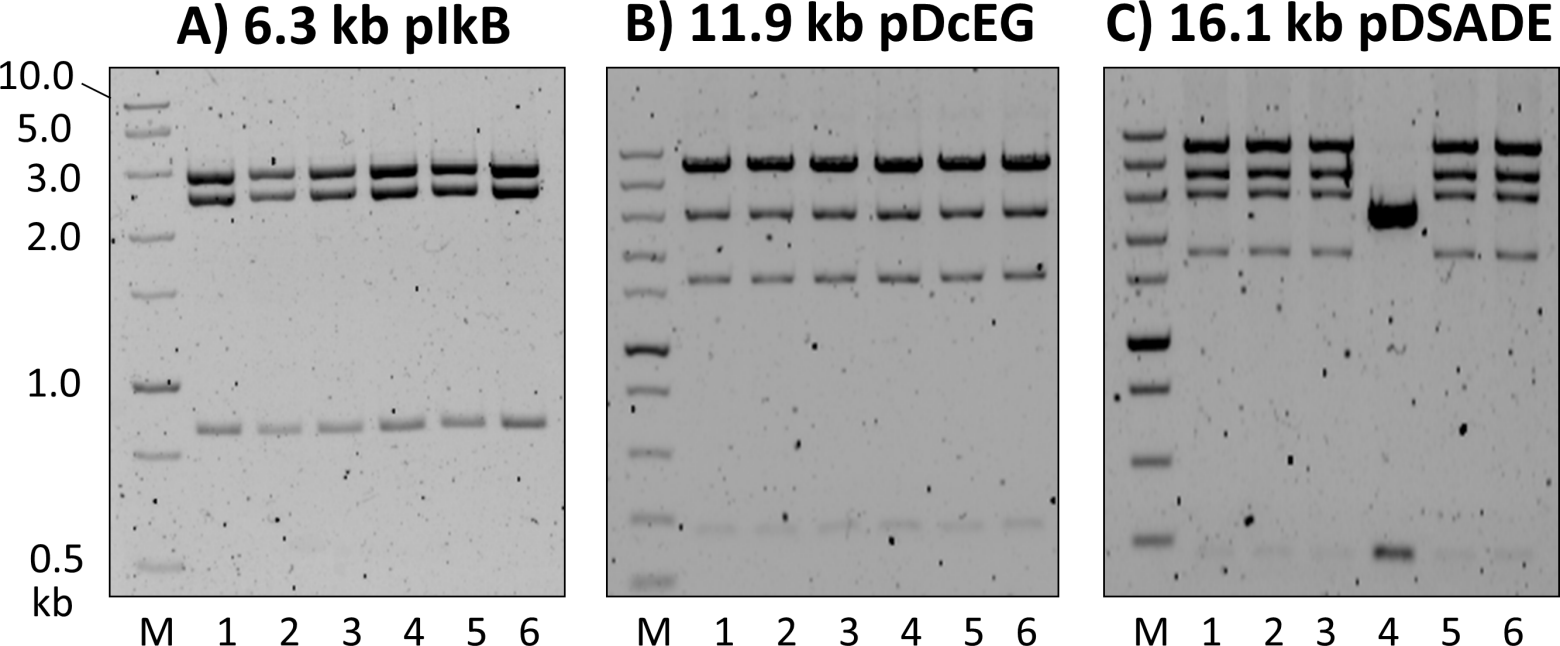
Confirmation of correct plasmid assembly by digestion with restriction enzymes. (**A**) pIkB digestion by SalI & NdeI resulting in 3 expected bands at 842, 2508, and 2979 bp, (**B**) pDcEG digestion by NsiI leading to 4 expected bands at 266, 1611, 2996, and 6940 bp, and (**C**) pDSADE digestion by NsiI generating 5 expected bands at 266, 1751, 2996, 4048, and 6940 bp. The 266 bp band was barely visible due to its expected weak intensity relative to other bands (only 3.8% of the 6940 bp band). No effort was made to identify the single negative plasmid (**lane 4** in **C**), but it was about 2.8 kb and contained KanR (the presence of the 266 bp signature band by NsiI digestion) as expected for growth under kanamycin conditions. It was not one of the two parental template plasmids (pDcEG & pDSA, S4 Table) used for PCR.

Cloning accuracy for the construction of pIkB, pDcEG, and pDSADE was determined by four different methods. First, colony PCR was performed on a panel of randomly selected colonies, followed by agarose gel electrophoresis analysis. Fig 8 shows the results from three panels performed on pIkB, pDcEG, and pDSADE assembly experiments, from which a combined 97% cloning accuracy (29 positives out of 30 colonies) was obtained. Second, plasmids from randomly chosen colonies were isolated. Restriction digestion and gel analysis were then performed to assess correctly assembled plasmids, as shown in Fig 9. While the number of isolated plasmids was limited, the results from Fig 9 gave high confidence of cloning accuracy by *in vivo* recombination (18/19 = 95%). It is worth noting that the combination of high colony number and easy visual detection of positive colonies for pGFP construction makes it highly reliable to calculate the cloning accuracy. While pIkB, pDcEG, and pDSADE assemblies had relative low colony numbers, the results from Figs 8 and 9 strongly suggest that *E*. *coli in vivo* assembly events by recombination have a 95% overall cloning accuracy irrespective of the number of DNA fragments, the length of overlapping ends, and the size of plasmids.

Intuitively, if a single recombination event has an accuracy of X (0< X <1), one would expect the overall cloning accuracy to be X^2^, X^3^, X^4^, and X^5^ for 2-, 3-, 4-, and 5-DNA fragment assemblies. Furthermore, one would presume that shorter homologous ends would lead to lower cloning accuracy. However, both predictions are inconsistent with our results. We believe that the predictions would be correct if the colony number would remain the same, but the decrease in colony number in both cases offsets the predicted decrease in cloning accuracy, resulting in an apparent constant cloning accuracy of 95% by *in vivo* assembly.

Third, to further confirm cloning accuracy, two isolated plasmids from each group of positive pGFP, pIkB, pDcEG, and pDSADE colonies were sequenced to cover all the joining regions by the Sanger method. No mutation, insertion, or deletion was observed. While positive results from green fluorescence screening, colony PCR, and restriction digestion did not exclude the possibility of mutations at single nucleotide resolution, Sanger sequencing gave definitive information that all positive colonies from screening had the expected sequences in all the joining regions, confirming that cloning through *E*. *coli in vivo* assembly is accurate.

Last, to assess the cloning accuracy of the whole process including PCR and *in vivo* assembly, one isolated plasmid from respective 2.6 kb pGFP and 16.1 kb pDSADE assemblies were whole plasmid-sequenced by NGS. The computer-generated plasmid sequences for both pGFP (S3 Sequence) (NGS raw data is accessible at NCBI SRA SRR5879307) and pDSADE (S6 Sequence) (NGS raw data is accessible at NCBI SRA SRR5879308) matched perfectly with the expected plasmid sequences. The results not only confirmed the high accuracy of *in vivo* assembly of homologous DNA fragments, but also validated our 2-consecutive PCR amplification of any DNA fragments up to 12 kb by Q5 DNA polymerase. While it is a common concern that extensive PCR amplification might introduce unintended mutations in DNA sequences, the results from whole plasmid-sequencing indicate that Q5 DNA polymerase can be used with high confidence to prepare quality DNA fragments (large or small, up to 12 kb) for *in vivo* cloning. Certain DNA sequences, such as the origin of replication, promoters, and polyadenylation sequences can be amplified extensively error-free with Q5 DNA polymerase. Considering that all the DNA fragments in pDSADE were PCR amplified and *in vivo* assembled through multiple rounds of cloning following the paths: pcDNA3.1/Hygro (+) → pcDNA3 Kan → pDcEG → pDSADE and pET28a-Ec.coaA + pET28a-Ec.coaD + pETite N-His SUMO → pDSA → pDSADE, it is remarkable that a randomly selected 16.1 kb target pDSADE has the exact pre-defined sequence without a single mutation. While the mechanism of *in vivo* cloning is still unknown [28], it requires homologous recombination activities that are independent of recA [31, 32]. However, the required homologous recombination activities do not interfere with stable plasmid DNA amplification in recA-negative *E*. *coli* cells, even when the plasmid contains repeating DNA sequences. The 16.1 kb pDSADE has 2 copies each of CGAAATTAATACGACTCACTATAGGG (26 nt, T7 promoter), AATTGTTATCCGCTCACAATTCC (23 nt, Lac operator), and GGTGTGGAAAGTCCCCAGGCTCCCCAGCAGGCAGAAGTATGCAAAGCATGCATCTCAATTAGTCAGCAACCA (72 nt, within SV 40 promoter). The isolated plasmid was correctly assembled according to the design (confirmed by whole plasmid sequencing), indicating that the homologous recombination activities act only on homologous DNA ends but not on internal repeating DNA sequences, which are stable against recombination.

While numerous cloning techniques have been developed–including the conventional restriction digestion and ligation [5, 6], LIC-PCR [7], SLIC [8], In-Fusion [9], USER [10], Gibson Assembly [11], Gateway [12], OEC [13, 14], SLiCE [15], and various *in vivo* homologous recombination methods [21–30], our cloning method is the simplest, fastest, and most economical. The only requirement is the DNA fragments with homologous ends prepared by PCR with the high fidelity Q5 DNA polymerase. Other *in vivo* assembly methods [26–28] involved ratio optimization of DNA fragments that were prepared separately, but we opted to use equal volumes of PCR reactions, eliminating the need for calculation and differential pipetting. Most existing cloning experiments use DpnI digestion to remove parental template plasmids, but complete digestion cannot be guaranteed, leading to a certain level of background colonies. In contrast, our 2-consecutive PCR procedure can easily bring the template plasmid concentration down to a level that results in zero background colony. The procedure is highly reliable and simple to carry out. The reported single-tube PCR preparation of multiple DNA fragments with homologous ends [29] is a simple method. However, it involves DpnI treatment of PCR products, and we had difficulties using this method to prepare DNA fragments for the construction of our plasmids.

While longer overlapping regions of DNA fragments can yield a higher number of colonies, longer DNA primers may increase the cost and the potential for mispriming. We believe that 25 nt OL represents the optimal balance for most cloning experiments. With the minimal volume of competent cells and PCR DNA fragments, our method is not optimized for high colony numbers. Instead, operational simplicity, high cloning accuracy, low costs, and short time for routine cloning were the primary goals of developing the method. With zero background and high reliability, low colony number is rarely a problem. For most routine cloning projects, a single colony containing accurately assembled plasmid is all that is needed. Typically, plasmid purification from 2 colonies on a transformation plate almost guarantees that at least one will have the correct target plasmid. We have often used isolated plasmids directly with high confidence in different projects, with their actual sequences being confirmed later by Sanger sequencing. On the other hand, if a research project demands a high number of colonies, increasing the length of DNA overlapping ends (OL from 25 nt to 40, 50, 60, 70 nt etc.) is the most efficient way, since the recombination efficiency increases exponentially with OL within the range [16]. In addition, the colony number can be further increased by using a larger volume of competent cells and DNA fragments (such as using 100 μL cells and 10 μL DNA mixture). However, the concentrations of template plasmids will need to be reduced further (e.g., 1 fg/μL) to maintain zero background from parental plasmids.

## Conclusions

A 2-consecutive PCR procedure using Q5 DNA polymerase has been developed to prepare high quality DNA fragments up to 12 kb and reduce template plasmid concentrations below 10 fg/μL. Directtransformation of a mixture of PCR DNA fragments containing overlapping ends into DH5α cells yields correctly assembled plasmids with a zero background from template plasmids. There is no need for colony screening, which is necessary in most cloning protocols. By analyzing *E*. *coli in vivo* assemblies of different plasmids under varying conditions, we have unraveled some intrinsic properties of homologous end recombination systems that are both recA- and Red/RecET-independent. While the *in vivo* cloning accuracy is maintained constant at 95% under different cloning conditions, the number of colonies increases with the length of overlapping nucleotides (Fig 4), but declines with the increasing number of DNA fragments (Fig 6) or the increasing size of target plasmids (Fig 7). Based on these properties, DNA fragments with 25 nt OL are recommended for general *in vivo* cloning, although shorter homologous ends with as low as 9 nt may be used for 2-DNA fragment assembly of small plasmids. Up to 5 DNA fragments may be used to construct small plasmids, while plasmids up to 16 kb can be assembled from 3 DNA fragments. Our *in vivo* cloning procedure is simple, fast, and economical for routine lab cloning, but it is not intended for complex DNA library construction.

## Supporting information

S1 Table DNA primers, template plasmids, and DNA fragments used for the construction of 2.6 kb pGFP. (PDF)

S2 Table DNA primers, template plasmids, and DNA fragments used for the construction of 6.3 kb pIkB. (PDF)

S3 Table DNA primers, template plasmids, and DNA fragments used for the construction of 11.9 kb pDcEG. (PDF)

S4 Table DNA primers, template plasmids, and DNA fragments used for the construction of 16.1 kb pDSADE. (PDF)

S1 Sequence 5.4 kb pcDNA3 Kan used as the backbone for the construction of pIkB, pDcEG, and pDSADE. (PDF)

S2 Sequence 7.0 kb pDSA used as the template for the construction of pDSADE. (PDF)

S3 Sequence 2.6 kb pGFP constructed by multiple DNA fragment assembly. (PDF)

S4 Sequence 6.3 kb pIkB constructed by multiple DNA fragment assembly. (PDF)

S5 Sequence 11.9 kb pDcEG constructed by multiple DNA fragment assembly. (PDF)

S6 Sequence 16.1 kb pDSADE constructed by multiple DNA fragment assembly. (PDF)
