# Supplementary material for: In vivo cloning of up to 16 kb plasmids in E. coli is as simple as PCR
Source: PLoS One. 2017 Aug 24;12(8):e0183974. doi: 10.1371/journal.pone.0183974 (PMC5570364; doi:10.1371/journal.pone.0183974)
Supplement: S1 Table — (PDF) [file pone.0183974.s001.pdf]

S1 Table. DNA fragments, templates, and primers for the construction of pGFP, 2,609 bp

nF	OL nt	No	Template	Primer pair sequence	DNA	Size bp
4F	18	F1	pTite N-His Kan	AACGAATTCAAGCTTGAT<br>CTCGATGAGTTTTTCTAATGAGGGCCCAAATG	Ori	791
		F2	pTite N-His Kan	TTAGAAAAACTCATCGAGC<br>GACGAATTCTCTAGATATCG	KanR	1003
		F3	pUC19	ATATCTAGAGAATTCGTCTAATGTGAGTTAGCTCACTC<br>TTCTCCTTTGCTAGCCATAGCTGTTTCCTGTGTG	P_{Lac}	146
		F4	pGLO	ATGGCTAGCAAAGGAGAA<br>ATCAAGCTTGAATTCGTTTCATTATTTGTAGAGCTCATC	GFP	741
2F	0	F1	pGFP	TATGGTGTTCAATGCTTTTCC<br>CTCGATGAGTTTTTCTAATGAGGGCCCAAATG	hGF-Ori	1316
		F2	pGFP	CATCAAGTGAAACTGCAATT<br>AGAGAAAGTAGTGACAAGTGT	KanR-P_{Lac}-hGF	1293
	6	F1	pGFP	ACTTTCTCTTATGGTGTTCAATGCTTTTCC<br>CTCGATGAGTTTTTCTAATGAGGGCCCAAATG	hGF-Ori	1325
		F2	pGFP	ATCGAGCATCAAGTGAAAC<br>GAAAGTAGTGACAAGTGTTG	KanR-P_{Lac}-hGF	1296
	9	F1	pGFP	ACTTTCTCTTATGGTGTTCAATGCTTTTCC<br>CTCGATGAGTTTTTCTAATGAGGGCCCAAATG	hGF-Ori	1325
		F2	pGFP	CTCATCGAGCATCAAGT<br>AGAGAAAGTAGTGACAAGTGT	KanR-P_{Lac}-hGF	1302
	12	F1	pGFP	ACTACTTTCTCTTATGGTGTTCAATGCTTTTCC<br>CTCGATGAGTTTTTCTAATGAGGGCCCAAATG	hGF-Ori	1328
		F2	pGFP	AAACTCATCGAGCATCA<br>AGAGAAAGTAGTGACAAGTGT	KanR-P_{Lac}-hGF	1305
	15	F1	pGFP	ACTACTTTCTCTTATGGTGTTCAATGCTTTTCC<br>CTCGATGAGTTTTTCTAATGAGGGCCCAAATG	hGF-Ori	1328
		F2	pGFP	GAAAAACTCATCGAGCAT<br>ATAAGAGAAAGTAGTGACAAGT	KanR-P_{Lac}-hGF	1311
	18	F1	pGFP	ACTACTTTCTCTTATGGTGTTCAATGCTTTTCC<br>CTCGATGAGTTTTTCTAATGAGGGCCCAAATG	hGF-Ori	1328
		F2	pGFP	TTAGAAAAACTCATCGAGC<br>ACCATAAGAGAAAGTAGTGAC	KanR-P_{Lac}-hGF	1317
	25	F1	pGFP	ACTACTTTCTCTTATGGTGTTCAATGCTTTTCC<br>CTCGATGAGTTTTTCTAATGAGGGCCCAAATG	hGF-Ori	1328
		F2	pGFP	GCCCTCATTAGAAAAACTCATCGAG<br>ATTGAACACCATAAGAGAAAGTAGTGACAAGTG	KanR-P_{Lac}-hGF	1331
3F	18	F1	pGFP	ACTACTTTCTCTTATGGTGTTCAATGCTTTTCC<br>ATCAAGCTTGAATTCGTTTCATTATTTGTAGAGCTCATC	hGF	555
		F2	pGFP	AACGAATTCAAGCTTGAT<br>CTCGATGAGTTTTTCTAATGAGGGCCCAAATG	Ori	791
		F3	pGFP	TTAGAAAAACTCATCGAGC<br>ACCATAAGAGAAAGTAGTGAC	KanR-P_{Lac}-hGF	1317
	25	F1	pGFP	ACTACTTTCTCTTATGGTGTTCAATGCTTTTCC<br>ATCAAGCTTGAATTCGTTTCATTATTTGTAGAGCTCATC	hGF	555
		F2	pGFP	ATAATGAAACGAATTCAAGCTTGAT<br>CTCGATGAGTTTTTCTAATGAGGGCCCAAATG	Ori	798
		F3	pGFP	GCCCTCATTAGAAAAACTCATCGAG<br>ATTGAACACCATAAGAGAAAGTAGTGACAAGTG	KanR-P_{Lac}-hGF	1331

4F	18	F1	pGFP	ACTACTTTCTCTTATGGTGTTCAATGCTTTTCC<br>ATCAAGCTTGAATTCGTTTCATTATTTGTAGAGCTCATC	hGF	555
		F2	pGFP	AACGAATTCAAGCTTGAT<br>CTCGATGAGTTTTTCTAATGAGGGCCCAAATG	Ori	791
		F3	pGFP	TTAGAAAAACTCATCGAGC<br>GACGAATTCTCTAGATATCG	KanR	1003
		F4	pGFP	ATATCTAGAGAATTCGTCTAATGTGAGTTAGCTCACTC<br>ACCATAAGAGAAAGTAGTGAC	P_{Lac}-hGF	332
	25	F1	pGFP	ACTACTTTCTCTTATGGTGTTCAATGCTTTTCC<br>ATCAAGCTTGAATTCGTTTCATTATTTGTAGAGCTCATC	hGF	555
		F2	pGFP	ATAATGAAACGAATTCAAGCTTGAT<br>CTCGATGAGTTTTTCTAATGAGGGCCCAAATG	Ori	798
		F3	pGFP	GCCCTCATTAGAAAAACTCATCGAG<br>CACATTAGACGAATTCTCTAGATAT	Kan	1017
		F4	pGFP	ATATCTAGAGAATTCGTCTAATGTGAGTTAGCTCACTC<br>ATTGAACACCATAAGAGAAAGTAGTGACAAGTG	P_{Lac}-hGF	339
5F	18	F1	pGFP	ACTACTTTCTCTTATGGTGTTCAATGCTTTTCC<br>ATCAAGCTTGAATTCGTTTCATTATTTGTAGAGCTCATC	hGF	555
		F2	pGFP	AACGAATTCAAGCTTGAT<br>CTCGATGAGTTTTTCTAATGAGGGCCCAAATG	Ori	791
		F3	pGFP	TTAGAAAAACTCATCGAGC<br>GACGAATTCTCTAGATATCG	Kan	1003
		F4	pGFP	ATATCTAGAGAATTCGTCTAATGTGAGTTAGCTCACTC<br>TTCTCCTTTGCTAGCCATAGCTGTTTCCTGTGTG	P_{Lac}	146
		F5	pGFP	ATGGCTAGCAAAGGAGAA<br>ACCATAAGAGAAAGTAGTGAC	hGF	204
	25	F1	pGFP	ACTACTTTCTCTTATGGTGTTCAATGCTTTTCC<br>ATCAAGCTTGAATTCGTTTCATTATTTGTAGAGCTCATC	hGF	555
		F2	pGFP	ATAATGAAACGAATTCAAGCTTGAT<br>CTCGATGAGTTTTTCTAATGAGGGCCCAAATG	Ori	798
		F3	pGFP	GCCCTCATTAGAAAAACTCATCGAG<br>CACATTAGACGAATTCTCTAGATAT	Kan	1017
		F4	pGFP	ATATCTAGAGAATTCGTCTAATGTGAGTTAGCTCACTC<br>TTCTCCTTTGCTAGCCATAGCTGTTTCCTGTGTG	P_{Lac}	146
		F5	pGFP	AACAGCTATGGCTAGCAAAGGAGAA<br>ATTGAACACCATAAGAGAAAGTAGTGACAAGTG	hGF	218

Notes:

1. **nF** = the number of DNA fragments used for plasmid assembly, e.g. **4F** = 4 fragments
2. OL (nt) = the number of overlapping nucleotides at the ends of DNA fragments
3. P_{Lac} = Lac promoter
4. Kan = kanamycin resistance gene
5. hGF = half GFP sequence
6. Complementary primer ends are color-coded among the multiple primers used to generate DNA fragments for the construction of pGFP. For example, the **5F**–25 nt OL system uses 10 primers to produce 5 DNA fragments with 5 pairs of matching ends. The 5 pairs of complementary primer ends with 25 nt are coded by 5 different colors. The same color cannot appear in the primer pair used to prepare a DNA fragment by PCR. Instead, the same-colored primer sequences must reside within the 5’ ends of the forward and reverse primers for neighboring DNA fragments.
