# Supplementary material for: In vivo cloning of up to 16 kb plasmids in E. coli is as simple as PCR
Source: PLoS One. 2017 Aug 24;12(8):e0183974. doi: 10.1371/journal.pone.0183974 (PMC5570364; doi:10.1371/journal.pone.0183974)
Supplement: S2 Table — (PDF) [file pone.0183974.s002.pdf]

S2 Table. DNA fragments, templates, and primers for the construction of pIkB, 6,332 bp

nF	OL nt	No	Template	Primer pair sequence	DNA	Size bp
**2F**	18	F1	pcDNA Kan	GCTGATCAGCCTCGACTG<br>GCAAGCTTAAGTTTAAACGCTAGC	Kan-Hyg-Ori-P_{CMV}	5343
		F2	pIK	GTTTAAACTTAAGCTTGCCACCATGGACTACAAAG<br>CAGTCGAGGCTGATCAGCGGGTTTATAACGTCAGACGCTG	IkB	1025
	25	F1	pcDNA Kan	TAAACCCGCTGATCAGCCTCGACTG<br>CATGGTGGCAAGCTTAAGTTTAAACGCTAGC	Kan-Hyg-Ori-P_{CMV}	5357
		F2	pIK	GTTTAAACTTAAGCTTGCCACCATGGACTACAAAG<br>CAGTCGAGGCTGATCAGCGGGTTTATAACGTCAGACGCTG	IkB	1025
**3F**	18	F1	pcDNA Kan	GCTGATCAGCCTCGACTG<br>CTCGATGAGTTTTTCTAACTGTCAGACCAAGTTTACTCAT	Hyg-Ori	3609
		F2	pcDNA Kan	TTAGAAAAACTCATCGAGC<br>GCAAGCTTAAGTTTAAACGCTAGC	Kan-P_{CMV}	1752
		F3	pIK	GTTTAAACTTAAGCTTGCCACCATGGACTACAAAG<br>CAGTCGAGGCTGATCAGCGGGTTTATAACGTCAGACGCTG	IkB	1025
	25	F1	pcDNA Kan	TAAACCCGCTGATCAGCCTCGACTG<br>CTCGATGAGTTTTTCTAACTGTCAGACCAAGTTTACTCAT	Hyg-Ori	3616
		F2	pcDNA Kan	CTGACAGTTAGAAAAACTCATCGAG<br>CATGGTGGCAAGCTTAAGTTTAAACGCTAGC	Kan-P_{CMV}	1766
		F3	pIK	GTTTAAACTTAAGCTTGCCACCATGGACTACAAAG<br>CAGTCGAGGCTGATCAGCGGGTTTATAACGTCAGACGCTG	IkB	1025

Note:

1. Hyg = hygromycin resistant gene
2. P_{CMV} = CMV promoter
3. IkB = NF-kappa-B inhibitor gene
