# Supplementary material for: In vivo cloning of up to 16 kb plasmids in E. coli is as simple as PCR
Source: PLoS One. 2017 Aug 24;12(8):e0183974. doi: 10.1371/journal.pone.0183974 (PMC5570364; doi:10.1371/journal.pone.0183974)
Supplement: S3 Table — (PDF) [file pone.0183974.s003.pdf]

S3 Table. DNA fragments, templates, and primers for the construction of pDcEG, 11,888 bp

nF	OL nt	No	Template	Primer pair sequence	DNA	Size bp
2F	18	F1	pcDNA Kan	TAAACCCGCTGATCAGCCT<br>GGCTTTTCATGGTGGCAAGCTTAAGTTTAAACGCTAGC	Kan-Hyg-Ori-P_{CMV}	5364
		F2	pDcEG	TTGCCACCATGAAAAGCCCTGCTTTGC<br>GGCTGATCAGCGGGTTTATTACTTGTACAGCTCGTC	Dcr-EGFP	6560
	25	F1	pcDNA Kan	TAAACCCGCTGATCAGCCTCGACTG<br>GGCTTTTCATGGTGGCAAGCTTAAGTTTAAACGCTAGC	Kan-Hyg-Ori-P_{CMV}	5364
		F2	pDcEG	CTTAAGCTTGCCACCATGAAAAGCCCTGCTTTGC<br>CAGTCGAGGCTGATCAGCGGGTTTATTACTTGTACAGCTCGTC	Dcr-EGFP	6574
3F	18	F1	pcDNA Kan	TAAACCCGCTGATCAGCCT<br>GGCTTTTCATGGTGGCAAGCTTAAGTTTAAACGCTAGC	Kan-Hyg-Ori-P_{CMV}	5364
		F2	Pk-Dcr	TTGCCACCATGAAAAGCCCTGCTTTGC<br>TCCAGCACTACCAGCGCTTCCGCTATTGGGAACC	Dcr	5795
		F3	pEGFP-N1	AGCGCTGGTAGTGCTGGAAGTGGTGAACCCAATAGCGCAGGTGCCATGGTGAGCAAGGG<br>GGCTGATCAGCGGGTTTATTACTTGTACAGCTCGTC	EGFP	783
	25	F1	pcDNA Kan	TAAACCCGCTGATCAGCCTCGACTG<br>GGCTTTTCATGGTGGCAAGCTTAAGTTTAAACGCTAGC	Kan-Hyg-Ori-P_{CMV}	5364
		F2	Pk-Dcr	CTTAAGCTTGCCACCATGAAAAGCCCTGCTTTGC<br>CACCACTTCCAGCACTACCAGCGCTTCCGCTATTGGGAACC	Dcr	5809
		F3	pEGFP-N1	AGCGCTGGTAGTGCTGGAAGTGGTGAACCCAATAGCGCAGGTGCCATGGTGAGCAAGGG<br>CAGTCGAGGCTGATCAGCGGGTTTATTACTTGTACAGCTCGTC	EGFP	790

Note: Dcr = human Dicer gene
