# Supplementary material for: In vivo cloning of up to 16 kb plasmids in E. coli is as simple as PCR
Source: PLoS One. 2017 Aug 24;12(8):e0183974. doi: 10.1371/journal.pone.0183974 (PMC5570364; doi:10.1371/journal.pone.0183974)
Supplement: S4 Table — (PDF) [file pone.0183974.s004.pdf]

S4 Table. DNA fragments, templates, and primers for the construction of pDSADE, 16,076 bp, 25 nt OL only

nF	No	Template	Primer pair sequence	DNA	Size bp
**2F**	F1	pDcEG	ACTTTGACATTGATTATTGACTAGTTATT<br>CCCCAAATCATACCTGACCTCCATA	Kan-Hyg-Ori-P_{CMV}-Dcr-EGFP	11795
	F2	pDSA	TATGGAGGTCAGGTATGATTTGGGGGTAATGATACCG<br>ACTAGTCAATAATCAATGTCAAAGTTCCTCCTTTCAGCAA	LacI-coaD-SUMO-CoaA	4331
**3F**	F1	pDcEG	ACTTTGACATTGATTATTGACTAGTTATT<br>CACCACTTCCAGCACTACCAGCGCTTCCGCTATTGGGAACC	P_{CMV}-Dcr	6487
	F2	pDcEG	AGCGCTGGTAGTGCTGGAAGTGGTGAACCCAATAGCGCAG<br>CCCCAAATCATACCTGACCTCCATA	EGFP-Kan-Hyg-Ori	5333
	F3	pDSA	TATGGAGGTCAGGTATGATTTGGGGGTAATGATACCG<br>ACTAGTCAATAATCAATGTCAAAGTTCCTCCTTTCAGCAA	LacI-coaD-SUMO-coaA	4331

Note:

1. coaA = coaA gene coding for *E. coli* pantothenate kinase
2. coaD = coaD gene coding for *E. coli* phosphopantetheine adenylyltransferase
