# Supplementary material for: In vivo cloning of up to 16 kb plasmids in E. coli is as simple as PCR
Source: PLoS One. 2017 Aug 24;12(8):e0183974. doi: 10.1371/journal.pone.0183974 (PMC5570364; doi:10.1371/journal.pone.0183974)
Supplement: S5 Sequence — (PDF) [file pone.0183974.s009.pdf]

S5 Sequence. pDcEG, 11888 bp

TTAGAAAAACTCATCGAGCATCAAGTGAAACTGCAATTTATTCATATCAGGATTATCAATACCATATTTTTGAAAAA
GCCGTTTCTGTAATGAAGGAGAAAACTCACCGAGGCAGTTCCATAGGATGGCAAGATCCTGGTATCGGTCTGCGATT
CCGACTCGTCCAACATCAATACAACCTATTAATTTCCCATCGTCAAAAATAAGGTTATCAAGTGAGAAATCACCATG
AGTGACGACTGAATCCGGTGAGAATGGCAAAAGCTTATGCATTTCTTTCCAGACTTGTTCAACAGGCCAGCCATTAC
GCTCGTCATCAAAATCACTCGCACCAACCAAACCGTTATTCATTCGTGATTGCGCCTGAGCGAGACGAAATACGCGA
TCGCCGTTAAAAGGACAATTACAAACAGGAATCGAATGCAACCGGCGCAGGAACACTGCCAGCGCATCAACAATATT
TTCACCTGAATCAGGATATTCTTCTAATACCTGGAATGCTGTTTTCCCTGGGATCGCAGTGGTGAGTAACCATGCAT
CATCAGGAGTACGGATAAAATGCTTGATGGTCGGAAGAGGCATAAATTCCGTCAGCCAGTTTAGCCTGACCATCTCA
TCTGTAACATCATTGGCAACGCTACCTTTGCCATGTTTCAGAAACAACTCTGGCGCATCGGGCTTCCCATACAATCG
ATAGATTGTCGCACCTGATTGCCCGACATTATCGCGAGCCCATTTATACCCATATAAATCAGCATCCATGTTGGAAT
TTAATCGCGGCCTCGAGCAAGACGTTTCCCGTTGAATATGGCTCATAGCTCCTGAAAATCTCGATAACTCAAAAAAT
ACGCCCGGTAGTGATCTTATTTCATTATGGTGAAAGTTGGAACCTCTTACGTGCCGATCAAGTCAAAAGCCTCCGGT
CGGAGGCTTTTGACTTTCTGCTATGGAGGTCAGGTATGATTTAAATGGTCAGTATTGAGCGATATCTAGAGAATTCG
TCGAAGAATCTGCTTAGGGTTAGGCGTTTTGCGCTGCTTCGCGATGTACGGGCCAGATATACGCGTTGACATTGATT
ATTGACTAGTTATTAATAGTAATCAATTACGGGGTCATTAGTTCATAGCCCATATATGGAGTTCCGCGTTACATAAC
TTACGGTAAATGGCCCGCCTGGCTGACCGCCCAACGACCCCCGCCCATTGACGTCAATAATGACGTATGTTCCCATA
GTAACGCCAATAGGGACTTTCCATTGACGTCAATGGGTGGAGTATTTACGGTAAACTGCCCACTTGGCAGTACATCA
AGTGTATCATATGCCAAGTACGCCCCCTATTGACGTCAATGACGGTAAATGGCCCGCCTGGCATTATGCCCAGTACA
TGACCTTATGGGACTTTCCTACTTGGCAGTACATCTACGTATTAGTCATCGCTATTACCATGGTGATGCGGTTTTGG
CAGTACATCAATGGGCGTGGATAGCGGTTTGACTCACGGGGATTTCCAAGTCTCCACCCCATTGACGTCAATGGGAG
TTTGTTTTGGCACCAAAATCAACGGGACTTTCCAAAATGTCGTAACAACTCCGCCCCATTGACGCAAATGGGCGGTA
GGCGTGTACGGTGGGAGGTCTATATAAGCAGAGCTCTCTGGCTAACTAGAGAACCCACTGCTTACTGGCTTATCGAA
ATTAATACGACTCACTATAGGGAGACCCAAGCTGGCTAGCGTTTAAACTTAAGCTTGCCACCATGAAAAGCCCTGCT
TTGCAACCCCTCAGCATGGCAGGCCTGCAGCTCATGACCCCTGCTTCCTCACCAATGGGTCCTTTCTTTGGACTGCC
ATGGCAACAAGAAGCAATTCATGATAACATTTATACGCCAAGAAAATATCAGGTTGAACTGCTTGAAGCAGCTCTGG
ATCATAATACCATCGTCTGTTTAAACACTGGCTCAGGGAAGACATTTATTGCAGTACTACTCACTAAAGAGCTGTCC
TATCAGATCAGGGGAGACTTCAGCAGAAATGGAAAAAGGACGGTGTTCTTGGTCAACTCTGCAAACCAGGTTGCTCA
ACAAGTGTCAGCTGTCAGAACTCATTCAGATCTCAAGGTTGGGGAATACTCAAACCTAGAAGTAAATGCATCTTGGA
CAAAAGAGAGATGGAACCAAGAGTTTACTAAGCACCAGGTTCTCATTATGACTTGCTATGTCGCCTTGAATGTTTTG
AAAAATGGTTACTTATCACTGTCAGACATTAACCTTTTGGTGTTTGATGAGTGTCATCTTGCAATCCTAGACCACCC
CTATCGAGAAATTATGAAGCTCTGTGAAAATTGTCCATCATGTCCTCGCATTTTGGGACTAACTGCTTCCATTTTAA
ATGGGAAATGTGATCCAGAGGAATTGGAAGAAAAGATTCAGAAACTAGAGAAAATTCTTAAGAGTAATGCTGAAACT
GCAACTGACCTGGTGGTCTTAGACAGGTATACTTCTCAGCCATGTGAGATTGTGGTGGATTGTGGACCATTTACTGA
CAGAAGTGGGCTTTATGAAAGACTGCTGATGGAATTAGAAGAAGCACTTAATTTTATCAATGATTGTAATATATCTG
TACATTCAAAAGAAAGAGATTCTACTTTAATTTCGAAACAGATACTATCAGACTGTCGTGCCGTATTGGTAGTTCTG
GGACCCTGGTGTGCAGATAAAGTAGCTGGAATGATGGTAAGAGAACTACAGAAATACATCAAACATGAGCAAGAGGA
GCTGCACAGGAAATTTTTATTGTTTACAGACACTTTCCTAAGGAAAATACATGCACTATGTGAAGAGCACTTCTCAC
CTGCCTCACTTGACCTGAAATTTGTAACTCCTAAAGTAATCAAACTGCTCGAAATCTTACGCAAATATAAACCATAT
GAGCGACAGCAGTTTGAAAGCGTTGAGTGGTATAATAATAGAAATCAGGATAATTATGTGTCATGGAGTGATTCTGA
GGATGATGATGAGGATGAAGAAATTGAAGAAAAAGAGAAGCCAGAGACAAATTTTCCTTCTCCTTTTACCAACATTT
TGTGCGGAATTATTTTTGTGGAAAGAAGATACACAGCAGTTGTCTTAAACAGATTGATAAAGGAAGCTGGCAAACAA
GATCCAGAGCTGGCTTATATCAGTAGCAATTTCATAACTGGACATGGCATTGGGAAGAATCAGCCTCGCAACAAACA
GATGGAAGCAGAATTCAGAAAACAGGAAGAGGTACTTAGGAAATTTCGAGCACATGAGACCAACCTGCTTATTGCAA
CAAGTATTGTAGAAGAGGGTGTTGATATACCAAAATGCAACTTGGTGGTTCGTTTTGATTTGCCCACAGAATATCGA
TCCTATGTTCAATCTAAAGGAAGAGCAAGGGCACCCATCTCTAATTATATAATGTTAGCGGATACAGACAAAATAAA
AAGTTTTGAAGAAGACCTTAAAACCTACAAAGCTATTGAAAAGATCTTGAGAAACAAGTGTTCCAAGTCGGTTGATA
CTGGTGAGACTGACATTGATCCTGTCATGGATGATGATGACGTTTTCCCACCATATGTGTTGAGGCCTGACGATGGT
GGTCCACGAGTCACAATCAACACGGCCATTGGACACATCAATAGATACTGTGCTAGATTACCAAGTGATCCGTTTAC
TCATCTAGCTCCTAAATGCAGAACCCGAGAGTTGCCTGATGGTACATTTTATTCAACTCTTTATCTGCCAATTAACT

CACCTCTTCGAGCCTCCATTGTTGGTCCACCAATGAGCTGTGTACGATTGGCTGAAAGAGTTGTAGCTCTCATTTGC
TGTGAGAAACTGCACAAAATTGGCGAACTGGATGACCATTTGATGCCAGTTGGGAAAGAGACTGTTAAATATGAAGA
GGAGCTTGATTTGCATGATGAAGAAGAGACCAGTGTTCCAGGAAGACCAGGTTCCACGAAACGAAGGCAGTGCTACC
CAAAAGCAATTCCAGAGTGTTTGAGGGATAGTTATCCCAGACCTGATCAGCCCTGTTACCTGTATGTGATAGGAATG
GTTTTAACTACACCTTTACCTGATGAACTCAACTTTAGAAGGCGGAAGCTCTATCCTCCTGAAGATACCACAAGATG
CTTTGGAATACTGACGGCCAAACCCATACCTCAGATTCCACACTTTCCTGTGTACACACGCTCTGGAGAGGTTACCA
TATCCATTGAGTTGAAGAAGTCTGGTTTCATGTTGTCTCTACAAATGCTTGAGTTGATTACAAGACTTCACCAGTAT
ATATTCTCACATATTCTTCGGCTTGAAAAACCTGCACTAGAATTTAAACCTACAGACGCTGATTCAGCATACTGTGT
TCTACCTCTTAATGTTGTTAATGACTCCAGCACTTTGGATATTGACTTTAAATTCATGGAAGATATTGAGAAGTCTG
AAGCTCGCATAGGCATTCCCAGTACAAAGTATACAAAAGAAACACCCTTTGTTTTTAAATTAGAAGATTACCAAGAT
GCCGTTATCATTCCAAGATATCGCAATTTTGATCAGCCTCATCGATTTTATGTAGCTGATGTGTACACTGATCTTAC
CCCACTCAGTAAATTTCCTTCCCCTGAGTATGAAACTTTTGCAGAATATTATAAAACAAAGTACAACCTTGACCTAA
CCAATCTCAACCAGCCACTGCTGGATGTGGACCACACATCTTCAAGACTTAATCTTTTGACACCTCGACATTTGAAT
CAGAAGGGGAAAGCGCTTCCTTTAAGCAGTGCTGAGAAGAGGAAAGCCAAATGGGAAAGTCTGCAGAATAAACAGAT
ACTGGTTCCAGAACTCTGTGCTATACATCCAATTCCAGCATCACTGTGGAGAAAAGCTGTTTGTCTCCCCAGCATAC
TTTATCGCCTTCACTGCCTTTTGACTGCAGAGGAGCTAAGAGCCCAGACTGCCAGCGATGCTGGCGTGGGAGTCAGA
TCACTTCCTGCGGATTTTAGATACCCTAACTTAGACTTCGGGTGGAAAAAATCTATTGACAGCAAATCTTTCATCTC
AATTTCTAACTCCTCTTCAGCTGAAAATGATAATTACTGTAAGCACAGCACAATTGTCCCTGAAAATGCTGCACATC
AAGGTGCTAATAGAACCTCCTCTCTAGAAAATCATGACCAAATGTCTGTGAACTGCAGAACGTTGCTCAGCGAGTCC
CCTGGTAAGCTCCACGTTGAAGTTTCAGCAGATCTTACAGCAATTAATGGTCTTTCTTACAATCAAAATCTCGCCAA
TGGCAGTTATGATTTAGCTAACAGAGACTTTTGCCAAGGAAATCAGCTAAATTACTACAAGCAGGAAATACCCGTGC
AACCAACTACCTCATATTCCATTCAGAATTTATACAGTTACGAGAACCAGCCCCAGCCCAGCGATGAATGTACTCTC
CTGAGTAATAAATACCTTGATGGAAATGCTAACAAATCTACCTCAGATGGAAGTCCTGTGATGGCCGTAATGCCTGG
TACGACAGACACTATTCAAGTGCTCAAGGGCAGGATGGATTCTGAGCAGAGCCCTTCTATTGGGTACTCCTCAAGGA
CTCTTGGCCCCAATCCTGGACTTATTCTTCAGGCTTTGACTCTGTCAAACGCTAGTGATGGATTTAACCTGGAGCGG
CTTGAAATGCTTGGCGACTCCTTTTTAAAGCATGCCATCACCACATATCTATTTTGCACTTACCCTGATGCGCATGA
GGGCCGCCTTTCATATATGAGAAGCAAAAAGGTCAGCAACTGTAATCTGTATCGCCTTGGAAAAAAGAAGGGACTAC
CCAGCCGCATGGTGGTGTCAATATTTGATCCCCCTGTGAATTGGCTTCCTCCTGGTTATGTAGTAAATCAAGACAAA
AGCAACACAGATAAATGGGAAAAAGATGAAATGACAAAAGACTGCATGCTGGCGAATGGCAAACTGGATGAGGATTA
CGAGGAGGAGGATGAGGAGGAGGAGAGCCTGATGTGGAGGGCTCCGAAGGAAGAGGCTGACTATGAAGATGATTTCC
TGGAGTATGATCAGGAACATATCAGATTTATAGATAATATGTTAATGGGGTCAGGAGCTTTTGTAAAGAAAATCTCT
CTTTCTCCTTTTTCAACCACTGATTCTGCATATGAATGGAAAATGCCCAAAAAATCCTCCTTAGGTAGTATGCCATT
TTCATCAGATTTTGAGGATTTTGACTACAGCTCTTGGGATGCAATGTGCTATCTGGATCCTAGCAAAGCTGTTGAAG
AAGATGACTTTGTGGTGGGGTTCTGGAATCCATCAGAAGAAAACTGTGGTGTTGACACGGGAAAGCAGTCCATTTCT
TACGACTTGCACACTGAGCAGTGTATTGCTGACAAAAGCATAGCGGACTGTGTGGAAGCCCTGCTGGGCTGCTATTT
AACCAGCTGTGGGGAGAGGGCTGCTCAGCTTTTCCTCTGTTCACTGGGGCTGAAGGTGCTCCCGGTAATTAAAAGGA
CTGATCGGGAAAAGGCCCTGTGCCCTACTCGGGAGAATTTCAACAGCCAACAAAAGAACCTTTCAGTGAGCTGTGCT
GCTGCTTCTGTGGCCAGTTCACGCTCTTCTGTATTGAAAGACTCGGAATATGGTTGTTTGAAGATTCCACCAAGATG
TATGTTTGATCATCCAGATGCAGATAAAACACTGAATCACCTTATATCGGGGTTTGAAAATTTTGAAAAGAAAATCA
ACTACAGATTCAAGAATAAGGCTTACCTTCTCCAGGCTTTTACACATGCCTCCTACCACTACAATACTATCACTGAT
TGTTACCAGCGCTTAGAATTCCTGGGAGATGCGATTTTGGACTACCTCATAACCAAGCACCTTTATGAAGACCCGCG
GCAGCACTCCCCGGGGGTCCTGACAGACCTGCGGTCTGCCCTGGTCAACAACACCATCTTTGCATCGCTGGCTGTAA
AGTACGACTACCACAAGTACTTCAAAGCTGTCTCTCCTGAGCTCTTCCATGTCATTGATGACTTTGTGCAGTTTCAG
CTTGAGAAGAATGAAATGCAAGGAATGGATTCTGAGCTTAGGAGATCTGAGGAGGATGAAGAGAAAGAAGAGGATAT
TGAAGTTCCAAAGGCCATGGGGGATATTTTTGAGTCGCTTGCTGGTGCCATTTACATGGATAGTGGGATGTCACTGG
AGACAGTCTGGCAGGTGTACTATCCCATGATGCGGCCACTAATAGAAAAGTTTTCTGCAAATGTACCCCGTTCCCCT
GTGCGAGAATTGCTTGAAATGGAACCAGAAACTGCCAAATTTAGCCCGGCTGAGAGAACTTACGACGGGAAGGTCAG
AGTCACTGTGGAAGTAGTAGGAAAGGGGAAATTTAAAGGTGTTGGTCGAAGTTACAGGATTGCCAAATCTGCAGCAG
CAAGAAGAGCCCTCCGAAGCCTCAAAGCTAATCAACCTCAGGTTCCCAATAGCGGAAGCGCTGGTAGTGCTGGAAGT
GGTGAACCCAATAGCGCAGGTGCCATGGTGAGCAAGGGCGAGGAGCTGTTCACCGGGGTGGTGCCCATCCTGGTCGA
GCTGGACGGCGACGTAAACGGCCACAAGTTCAGCGTGTCCGGCGAGGGCGAGGGCGATGCCACCTACGGCAAGCTGA
CCCTGAAGTTCATCTGCACCACCGGCAAGCTGCCCGTGCCCTGGCCCACCCTCGTGACCACCCTGACCTACGGCGTG
CAGTGCTTCAGCCGCTACCCCGACCACATGAAGCAGCACGACTTCTTCAAGTCCGCCATGCCCGAAGGCTACGTCCA

GGAGCGCACCATCTTCTTCAAGGACGACGGCAACTACAAGACCCGCGCCGAGGTGAAGTTCGAGGGCGACACCCTGG
TGAACCGCATCGAGCTGAAGGGCATCGACTTCAAGGAGGACGGCAACATCCTGGGGCACAAGCTGGAGTACAACTAC
AACAGCCACAACGTCTATATCATGGCCGACAAGCAGAAGAACGGCATCAAGGTGAACTTCAAGATCCGCCACAACAT
CGAGGACGGCAGCGTGCAGCTCGCCGACCACTACCAGCAGAACACCCCCATCGGCGACGGCCCCGTGCTGCTGCCCG
ACAACCACTACCTGAGCACCCAGTCCGCCCTGAGCAAAGACCCCAACGAGAAGCGCGATCACATGGTCCTGCTGGAG
TTCGTGACCGCCGCCGGGATCACTCTCGGCATGGACGAGCTGTACAAGTAATAAACCCGCTGATCAGCCTCGACTGT
GCCTTCTAGTTGCCAGCCATCTGTTGTTTGCCCCTCCCCCGTGCCTTCCTTGACCCTGGAAGGTGCCACTCCCACTG
TCCTTTCCTAATAAAATGAGGAAATTGCATCGCATTGTCTGAGTAGGTGTCATTCTATTCTGGGGGGTGGGGTGGGG
CAGGACAGCAAGGGGGAGGATTGGGAAGACAATAGCAGGCATGCTGGGGATGCGGTGGGCTCTATGGCTTCTGAGGC
GGAAAGAACCAGCTGGGGCTCTAGGGGGTATCCCCACGCGCCCTGTAGCGGCGCATTAAGCGCGGCGGGTGTGGTGG
TTACGCGCAGCGTGACCGCTACACTTGCCAGCGCCCTAGCGCCCGCTCCTTTCGCTTTCTTCCCTTCCTTTCTCGCC
ACGTTCGCCGGCTTTCCCCGTCAAGCTCTAAATCGGGGGCTCCCTTTAGGGTTCCGATTTAGTGCTTTACGGCACCT
CGACCCCAAAAAACTTGATTAGGGTGATGGTTCACGTAGTGGGCCATCGCCCTGATAGACGGTTTTTCGCCCTTTGA
CGTTGGAGTCCACGTTCTTTAATAGTGGACTCTTGTTCCAAACTGGAACAACACTCAACCCTATCTCGGTCTATTCT
TTTGATTTATAAGGGATTTTGCCGATTTCGGCCTATTGGTTAAAAAATGAGCTGATTTAACAAAAATTTAACGCGAA
TTAATTCTGTGGAATGTGTGTCAGTTAGGGTGTGGAAAGTCCCCAGGCTCCCCAGCAGGCAGAAGTATGCAAAGCAT
GCATCTCAATTAGTCAGCAACCAGGTGTGGAAAGTCCCCAGGCTCCCCAGCAGGCAGAAGTATGCAAAGCATGCATC
TCAATTAGTCAGCAACCATAGTCCCGCCCCTAACTCCGCCCATCCCGCCCCTAACTCCGCCCAGTTCCGCCCATTCT
CCGCCCCATGGCTGACTAATTTTTTTTATTTATGCAGAGGCCGAGGCCGCCTCTGCCTCTGAGCTATTCCAGAAGTA
GTGAGGAGGCTTTTTTGGAGGCCTAGGCTTTTGCAAAAAGCTCCCGGGAGCTTGTATATCCATTTTCGGATCTGATC
AGCACGTGATGAAAAAGCCTGAACTCACCGCGACGTCTGTCGAGAAGTTTCTGATCGAAAAGTTCGACAGCGTCTCC
GACCTGATGCAGCTCTCGGAGGGCGAAGAATCTCGTGCTTTCAGCTTCGATGTAGGAGGGCGTGGATATGTCCTGCG
GGTAAATAGCTGCGCCGATGGTTTCTACAAAGATCGTTATGTTTATCGGCACTTTGCATCGGCCGCGCTCCCGATTC
CGGAAGTGCTTGACATTGGGGAATTCAGCGAGAGCCTGACCTATTGCATCTCCCGCCGTGCACAGGGTGTCACGTTG
CAAGACCTGCCTGAAACCGAACTGCCCGCTGTTCTGCAGCCGGTCGCGGAGGCCATGGATGCGATCGCTGCGGCCGA
TCTTAGCCAGACGAGCGGGTTCGGCCCATTCGGACCGCAAGGAATCGGTCAATACACTACATGGCGTGATTTCATAT
GCGCGATTGCTGATCCCCATGTGTATCACTGGCAAACTGTGATGGACGACACCGTCAGTGCGTCCGTCGCGCAGGCT
CTCGATGAGCTGATGCTTTGGGCCGAGGACTGCCCCGAAGTCCGGCACCTCGTGCACGCGGATTTCGGCTCCAACAA
TGTCCTGACGGACAATGGCCGCATAACAGCGGTCATTGACTGGAGCGAGGCGATGTTCGGGGATTCCCAATACGAGG
TCGCCAACATCTTCTTCTGGAGGCCGTGGTTGGCTTGTATGGAGCAGCAGACGCGCTACTTCGAGCGGAGGCATCCG
GAGCTTGCAGGATCGCCGCGGCTCCGGGCGTATATGCTCCGCATTGGTCTTGACCAACTCTATCAGAGCTTGGTTGA
CGGCAATTTCGATGATGCAGCTTGGGCGCAGGGTCGATGCGACGCAATCGTCCGATCCGGAGCCGGGACTGTCGGGC
GTACACAAATCGCCCGCAGAAGCGCGGCCGTCTGGACCGATGGCTGTGTAGAAGTACTCGCCGATAGTGGAAACCGA
CGCCCCAGCACTCGTCCGAGGGCAAAGGAATAGCACGTGCTACGAGATTTCGATTCCACCGCCGCCTTCTATGAAAG
GTTGGGCTTCGGAATCGTTTTCCGGGACGCCGGCTGGATGATCCTCCAGCGCGGGGATCTCATGCTGGAGTTCTTCG
CCCACCCCAACTTGTTTATTGCAGCTTATAATGGTTACAAATAAAGCAATAGCATCACAAATTTCACAAATAAAGCA
TTTTTTTCACTGCATTCTAGTTGTGGTTTGTCCAAACTCATCAATGTATCTTATCATGTCTGTATACCGTCGACCTC
TAGCTAGAGCTTGGCGTAATCATGGTCATAGCTGTTTCCTGTGTGAAATTGTTATCCGCTCACAATTCCACACAACA
TACGAGCCGGAAGCATAAAGTGTAAAGCCTGGGGTGCCTAATGAGTGAGCTAACTCACATTAATTGCGTTGCGCTCA
CTGCCCGCTTTCCAGTCGGGAAACCTGTCGTGCCAGCTGCATTAATGAATCGGCCAACGCGCGGGGAGAGGCGGTTT
GCGTATTGGGCGCTCTTCCGCTTCCTCGCTCACTGACTCGCTGCGCTCGGTCGTTCGGCTGCGGCGAGCGGTATCAG
CTCACTCAAAGGCGGTAATACGGTTATCCACAGAATCAGGGGATAACGCAGGAAAGAACATGTGAGCAAAAGGCCAG
CAAAAGGCCAGGAACCGTAAAAAGGCCGCGTTGCTGGCGTTTTTCCATAGGCTCCGCCCCCCTGACGAGCATCACAA
AAATCGACGCTCAAGTCAGAGGTGGCGAAACCCGACAGGACTATAAAGATACCAGGCGTTTCCCCCTGGAAGCTCCC
TCGTGCGCTCTCCTGTTCCGACCCTGCCGCTTACCGGATACCTGTCCGCCTTTCTCCCTTCGGGAAGCGTGGCGCTT
TCTCATAGCTCACGCTGTAGGTATCTCAGTTCGGTGTAGGTCGTTCGCTCCAAGCTGGGCTGTGTGCACGAACCCCC
CGTTCAGCCCGACCGCTGCGCCTTATCCGGTAACTATCGTCTTGAGTCCAACCCGGTAAGACACGACTTATCGCCAC
TGGCAGCAGCCACTGGTAACAGGATTAGCAGAGCGAGGTATGTAGGCGGTGCTACAGAGTTCTTGAAGTGGTGGCCT
AACTACGGCTACACTAGAAGAACAGTATTTGGTATCTGCGCTCTGCTGAAGCCAGTTACCTTCGGAAAAAGAGTTGG
TAGCTCTTGATCCGGCAAACAAACCACCGCTGGTAGCGGTGGTTTTTTTGTTTGCAAGCAGCAGATTACGCGCAGAA
AAAAAGGATCTCAAGAAGATCCTTTGATCTTTTCTACGGGGTCTGACGCTCAGTGGAACGAAAACTCACGTTAAGGG
ATTTTGGTCATGAGATTATCAAAAAGGATCTTCACCTAGATCCTTTTAAATTAAAAATGAAGTTTTAAATCAATCTA
AAGTATATATGAGTAAACTTGGTCTGACAG
