# Supplementary material for: In vivo cloning of up to 16 kb plasmids in E. coli is as simple as PCR
Source: PLoS One. 2017 Aug 24;12(8):e0183974. doi: 10.1371/journal.pone.0183974 (PMC5570364; doi:10.1371/journal.pone.0183974)
Supplement: S6 Sequence — (PDF) [file pone.0183974.s010.pdf]

S6 Sequence. pDSADE, 16076 bp

TTAGAAAAACTCATCGAGCATCAAGTGAAACTGCAATTTATTCATATCAGGATTATCAATACCATATTTTTGAAAAA
GCCGTTTCTGTAATGAAGGAGAAAACTCACCGAGGCAGTTCCATAGGATGGCAAGATCCTGGTATCGGTCTGCGATT
CCGACTCGTCCAACATCAATACAACCTATTAATTTCCCATCGTCAAAAATAAGGTTATCAAGTGAGAAATCACCATG
AGTGACGACTGAATCCGGTGAGAATGGCAAAAGCTTATGCATTTCTTTCCAGACTTGTTCAACAGGCCAGCCATTAC
GCTCGTCATCAAAATCACTCGCACCAACCAAACCGTTATTCATTCGTGATTGCGCCTGAGCGAGACGAAATACGCGA
TCGCCGTTAAAAGGACAATTACAAACAGGAATCGAATGCAACCGGCGCAGGAACACTGCCAGCGCATCAACAATATT
TTCACCTGAATCAGGATATTCTTCTAATACCTGGAATGCTGTTTTCCCTGGGATCGCAGTGGTGAGTAACCATGCAT
CATCAGGAGTACGGATAAAATGCTTGATGGTCGGAAGAGGCATAAATTCCGTCAGCCAGTTTAGCCTGACCATCTCA
TCTGTAACATCATTGGCAACGCTACCTTTGCCATGTTTCAGAAACAACTCTGGCGCATCGGGCTTCCCATACAATCG
ATAGATTGTCGCACCTGATTGCCCGACATTATCGCGAGCCCATTTATACCCATATAAATCAGCATCCATGTTGGAAT
TTAATCGCGGCCTCGAGCAAGACGTTTCCCGTTGAATATGGCTCATAGCTCCTGAAAATCTCGATAACTCAAAAAAT
ACGCCCGGTAGTGATCTTATTTCATTATGGTGAAAGTTGGAACCTCTTACGTGCCGATCAAGTCAAAAGCCTCCGGT
CGGAGGCTTTTGACTTTCTGCTATGGAGGTCAGGTATGATTTGGGGGTAATGATACCGATGAAACGAGAGAGGATGC
TCACGATACGGGTTACTGATGATGAACATGCCCGGTTACTGGAACGTTGTGAGGGTAAACAACTGGCGGTATGGATG
CGGCGGGACCAGAGAAAAATCACTCAGGGTCAATGCCAGCGCTTCGTTAATACAGATGTAGGTGTTCCACAGGGTAG
CCAGCAGCATCCTGCGATGCAGATCCGGAACATAATGGTGCAGGGCGCTGACTTCCGCGTTTCCAGACTTTACGAAA
CACGGAAACCGAAGACCATTCATGTTGTTGCTCAGGTCGCAGACGTTTTGCAGCAGCAGTCGCTTCACGTTCGCTCG
CGTATCGGTGATTCATTCTGCTAACCAGTAAGGCAACCCCGCCAGCCTAGCCGGGTCCTCAACGACAGGAGCACGAT
CATGCGCACCCGTGGGGCCGCCATGCCGGCGATAATGGCCTGCTTCTCGCCGAAACGTTTGGTGGCGGGACCAGTGA
CGAAGGCTTGAGCGAGGGCGTGCAAGATTCCGAATACCGCAAGCGACAGGCCGATCATCGTCGCGCTCCAGCGAAAG
CGGTCCTCGCCGAAAATGACCCAGAGCGCTGCCGGCACCTGTCCTACGAGTTGCATGATAAAGAAGACAGTCATAAG
TGCGGCGACGATAGTCATGCCCCGCGCCCACCGGAAGGAGCTGACTGGGTTGAAGGCTCTCAAGGGCATCGGTCGAG
ATCCCGGTGCCTAATGAGTGAGCTAACTTACATTAATTGCGTTGCGCTCACTGCCCGCTTTCCAGTCGGGAAACCTG
TCGTGCCAGCTGCATTAATGAATCGGCCAACGCGCGGGGAGAGGCGGTTTGCGTATTGGGCGCCAGGGTGGTTTTTC
TTTTCACCAGTGAGACGGGCAACAGCTGATTGCCCTTCACCGCCTGGCCCTGAGAGAGTTGCAGCAAGCGGTCCACG
CTGGTTTGCCCCAGCAGGCGAAAATCCTGTTTGATGGTGGTTAACGGCGGGATATAACATGAGCTGTCTTCGGTATC
GTCGTATCCCACTACCGAGATATCCGCACCAACGCGCAGCCCGGACTCGGTAATGGCGCGCATTGCGCCCAGCGCCA
TCTGATCGTTGGCAACCAGCATCGCAGTGGGAACGATGCCCTCATTCAGCATTTGCATGGTTTGTTGAAAACCGGAC
ATGGCACTCCAGTCGCCTTCCCGTTCCGCTATCGGCTGAATTTGATTGCGAGTGAGATATTTATGCCAGCCAGCCAG
ACGCAGACGCGCCGAGACAGAACTTAATGGGCCCGCTAACAGCGCGATTTGCTGGTGACCCAATGCGACCAGATGCT
CCACGCCCAGTCGCGTACCGTCTTCATGGGAGAAAATAATACTGTTGATGGGTGTCTGGTCAGAGACATCAAGAAAT
AACGCCGGAACATTAGTGCAGGCAGCTTCCACAGCAATGGCATCCTGGTCATCCAGCGGATAGTTAATGATCAGCCC
ACTGACGCGTTGCGCGAGAAGATTGTGCACCGCCGCTTTACAGGCTTCGACGCCGCTTCGTTCTACCATCGACACCA
CCACGCTGGCACCCAGTTGATCGGCGCGAGATTTAATCGCCGCGACAATTTGCGACGGCGCGTGCAGGGCCAGACTG
GAGGTGGCAACGCCAATCAGCAACGACTGTTTGCCCGCCAGTTGTTGTGCCACGCGGTTGGGAATGTAATTCAGCTC
CGCCATCGCCGCTTCCACTTTTTCCCGCGTTTTCGCAGAAACGTGGCTGGCCTGGTTCACCACGCGGGAAACGGTCT
GATAAGAGACACCGGCATACTCTGCGACATCGTATAACGTTACTGGTTTCACATTCACCACCCTGAATTGACTCTCT
TCCGGGCGCTATCATGCCATACCGCGAAAGGTTTTGCGCCATTCGATGGTGTCCGGGATCTCGACGCTCTCCCTTAT
GCGACTCCTGCATTAGGAAGCAGCCCAGTAGTAGGTTGAGGCCGTTGAGCACCGCCGCCGCAAGGAATGGTGCATGC
AAGGAGATGGCGCCCAACAGTCCCCCGGCCACGGGGCCTGCCACCATACCCACGCCGAAACAAGCGCTCATGAGCCC
GAAGTGGCGAGCCCGATCTTCCCCATCGGTGATGTCGGCGATATAGGCGCCAGCAACCGCACCTGTGGCGCCGGTGA
TGCCGGCCACGATGCGTCCGGCGTAGAGGATCGAGATCTCGATCCCGCGAAATTAATACGACTCACTATAGGGGAAT
TGTGAGCGGATAACAATTCCCCTCTAGAAATAATTTTGTTTAACTTTAAGAAGGAGATATACCATGGGCAGCAGCCA
TCATCATCATCATCACAGCAGCGGCCTGGTGCCGCGCGGCAGCCATATGCAAAAACGGGCGATTTATCCGGGTACTT
TCGATCCCATTACCAATGGTCATATCGATATCGTGACGCGCGCCACGCAGATGTTCGATCACGTTATTCTGGCGATT
GCCGCCAGCCCCAGTAAAAAACCGATGTTTACCCTGGAAGAGCGTGTGGCACTGGCACAGCAGGCAACCGCGCATCT
GGGGAACGTGGAAGTGGTCGGGTTTAGTGATTTAATGGCGAACTTCGCCCGTAATCAACACGCTACGGTGCTGATTC
GTGGCCTGCGTGCGGTGGCAGATTTTGAATATGAAATGCAGCTGGCGCACATGAATCGCCACTTAATGCCGGAACTG
GAAAGTGTGTTTCTGATGCCGTCGAAAGAGTGGTCGTTTATCTCTTCATCGTTGGTGAAAGAGGTGGCGCGCCATCA

GGGCGATGTCACCCATTTCCTGCCGGAGAATGTCCATCAGGCGCTGATGGCGAAGTTAGCGCAGGACTCAGAAGTCA
ATCAAGAAGCTAAGCCAGAGGTCAAGCCAGAAGTCAAGCCTGAGACTCACATCAATTTAAAGGTGTCCGATGGATCT
TCAGAGATCTTCTTCAAGATCAAAAAGACCACTCCTTTAAGAAGGCTGATGGAAGCGTTCGCTAAAAGACAGGGTAA
GGAAATGGACTCCTTAACGTTCTTGTACGACGGTATTGAAATTCAAGCTGATCAGACCCCTGAAGATTTGGACATGG
AGGATAACGATATTATTGAGGCTCACCGCGAACAGATTGGAGGTATGAGTATAAAAGAGCAAACGTTAATGACGCCT
TACCTACAGTTTGACCGCAACCAGTGGGCAGCTCTGCGTGATTCCGTACCTATGACGTTATCGGAAGATGAGATCGC
CCGTCTCAAAGGTATTAATGAAGATCTCTCGTTAGAAGAAGTTGCCGAGATCTATTTACCTTTGTCACGTTTGCTGA
ACTTCTATATAAGCTCGAATCTGCGCCGTCAGGCAGTTCTGGAACAGTTTCTTGGTACTAACGGGCAACGCATTCCT
TACATTATCAGTATTGCTGGCAGTGTCGCGGTGGGGAAAAGTACAACCGCCCGTGTATTGCAGGCGCTATTAAGCCG
TTGGCCGGAACATCGTCGTGTTGAACTGATCACTACAGATGGCTTCCTTCACCCTAATCAGGTTCTGAAAGAACGTG
GTCTGATGAAGAAGAAAGGCTTCCCGGAATCGTATGATATGCATCGCCTGGTGAAGTTTGTTTCCGATCTCAAATCC
GGCGTGCCAAACGTTACAGCCCCTGTTTACTCGCATCTTATTTATGATGTGATCCCGGATGGAGATAAAACGGTTGT
TCAGCCTGATATTTTAATTCTTGAAGGGTTAAATGTCTTACAGAGCGGGATGGATTATCCACACGATCCACATCATG
TATTTGTTTCTGATTTTGTCGATTTTTCGATATATGTTGATGCACCGGAAGACTTACTTCAGACGTGGTATATCAAC
CGTTTTCTGAAATTCCGCGAAGGGGCTTTTACCGACCCGGATTCCTATTTTCATAACTACGCGAAATTAACTAAAGA
AGAAGCGATTAAGACTGCCATGACATTGTGGAAAGAGATCAACTGGCTGAACTTAAAGCAAAATATTCTACCTACTC
GTGAGCGCGCCAGTTTAATCCTGACGAAAAGTGCTAATCATGCGGTAGAAGAGGTCAGACTACGCAAATAACTCCGT
CGACAAGCTTGCGGCCGCACTCGAGCACCACCACCACCACCACTGAGATCCGGCTGCTAACAAAGCCCGAAAGGAAG
CTGAGTTGGCTGCTGCCACCGCTGAGCAATAACTAGCATAACCCCTTGGGGCCTCTAAACGGGTCTTGAGGGGTTTT
TTGCTGAAAGGAGGAACTTTGACATTGATTATTGACTAGTTATTAATAGTAATCAATTACGGGGTCATTAGTTCATA
GCCCATATATGGAGTTCCGCGTTACATAACTTACGGTAAATGGCCCGCCTGGCTGACCGCCCAACGACCCCCGCCCA
TTGACGTCAATAATGACGTATGTTCCCATAGTAACGCCAATAGGGACTTTCCATTGACGTCAATGGGTGGAGTATTT
ACGGTAAACTGCCCACTTGGCAGTACATCAAGTGTATCATATGCCAAGTACGCCCCCTATTGACGTCAATGACGGTA
AATGGCCCGCCTGGCATTATGCCCAGTACATGACCTTATGGGACTTTCCTACTTGGCAGTACATCTACGTATTAGTC
ATCGCTATTACCATGGTGATGCGGTTTTGGCAGTACATCAATGGGCGTGGATAGCGGTTTGACTCACGGGGATTTCC
AAGTCTCCACCCCATTGACGTCAATGGGAGTTTGTTTTGGCACCAAAATCAACGGGACTTTCCAAAATGTCGTAACA
ACTCCGCCCCATTGACGCAAATGGGCGGTAGGCGTGTACGGTGGGAGGTCTATATAAGCAGAGCTCTCTGGCTAACT
AGAGAACCCACTGCTTACTGGCTTATCGAAATTAATACGACTCACTATAGGGAGACCCAAGCTGGCTAGCGTTTAAA
CTTAAGCTTGCCACCATGAAAAGCCCTGCTTTGCAACCCCTCAGCATGGCAGGCCTGCAGCTCATGACCCCTGCTTC
CTCACCAATGGGTCCTTTCTTTGGACTGCCATGGCAACAAGAAGCAATTCATGATAACATTTATACGCCAAGAAAAT
ATCAGGTTGAACTGCTTGAAGCAGCTCTGGATCATAATACCATCGTCTGTTTAAACACTGGCTCAGGGAAGACATTT
ATTGCAGTACTACTCACTAAAGAGCTGTCCTATCAGATCAGGGGAGACTTCAGCAGAAATGGAAAAAGGACGGTGTT
CTTGGTCAACTCTGCAAACCAGGTTGCTCAACAAGTGTCAGCTGTCAGAACTCATTCAGATCTCAAGGTTGGGGAAT
ACTCAAACCTAGAAGTAAATGCATCTTGGACAAAAGAGAGATGGAACCAAGAGTTTACTAAGCACCAGGTTCTCATT
ATGACTTGCTATGTCGCCTTGAATGTTTTGAAAAATGGTTACTTATCACTGTCAGACATTAACCTTTTGGTGTTTGA
TGAGTGTCATCTTGCAATCCTAGACCACCCCTATCGAGAAATTATGAAGCTCTGTGAAAATTGTCCATCATGTCCTC
GCATTTTGGGACTAACTGCTTCCATTTTAAATGGGAAATGTGATCCAGAGGAATTGGAAGAAAAGATTCAGAAACTA
GAGAAAATTCTTAAGAGTAATGCTGAAACTGCAACTGACCTGGTGGTCTTAGACAGGTATACTTCTCAGCCATGTGA
GATTGTGGTGGATTGTGGACCATTTACTGACAGAAGTGGGCTTTATGAAAGACTGCTGATGGAATTAGAAGAAGCAC
TTAATTTTATCAATGATTGTAATATATCTGTACATTCAAAAGAAAGAGATTCTACTTTAATTTCGAAACAGATACTA
TCAGACTGTCGTGCCGTATTGGTAGTTCTGGGACCCTGGTGTGCAGATAAAGTAGCTGGAATGATGGTAAGAGAACT
ACAGAAATACATCAAACATGAGCAAGAGGAGCTGCACAGGAAATTTTTATTGTTTACAGACACTTTCCTAAGGAAAA
TACATGCACTATGTGAAGAGCACTTCTCACCTGCCTCACTTGACCTGAAATTTGTAACTCCTAAAGTAATCAAACTG
CTCGAAATCTTACGCAAATATAAACCATATGAGCGACAGCAGTTTGAAAGCGTTGAGTGGTATAATAATAGAAATCA
GGATAATTATGTGTCATGGAGTGATTCTGAGGATGATGATGAGGATGAAGAAATTGAAGAAAAAGAGAAGCCAGAGA
CAAATTTTCCTTCTCCTTTTACCAACATTTTGTGCGGAATTATTTTTGTGGAAAGAAGATACACAGCAGTTGTCTTA
AACAGATTGATAAAGGAAGCTGGCAAACAAGATCCAGAGCTGGCTTATATCAGTAGCAATTTCATAACTGGACATGG
CATTGGGAAGAATCAGCCTCGCAACAAACAGATGGAAGCAGAATTCAGAAAACAGGAAGAGGTACTTAGGAAATTTC
GAGCACATGAGACCAACCTGCTTATTGCAACAAGTATTGTAGAAGAGGGTGTTGATATACCAAAATGCAACTTGGTG
GTTCGTTTTGATTTGCCCACAGAATATCGATCCTATGTTCAATCTAAAGGAAGAGCAAGGGCACCCATCTCTAATTA
TATAATGTTAGCGGATACAGACAAAATAAAAAGTTTTGAAGAAGACCTTAAAACCTACAAAGCTATTGAAAAGATCT
TGAGAAACAAGTGTTCCAAGTCGGTTGATACTGGTGAGACTGACATTGATCCTGTCATGGATGATGATGACGTTTTC
CCACCATATGTGTTGAGGCCTGACGATGGTGGTCCACGAGTCACAATCAACACGGCCATTGGACACATCAATAGATA

CTGTGCTAGATTACCAAGTGATCCGTTTACTCATCTAGCTCCTAAATGCAGAACCCGAGAGTTGCCTGATGGTACAT
TTTATTCAACTCTTTATCTGCCAATTAACTCACCTCTTCGAGCCTCCATTGTTGGTCCACCAATGAGCTGTGTACGA
TTGGCTGAAAGAGTTGTAGCTCTCATTTGCTGTGAGAAACTGCACAAAATTGGCGAACTGGATGACCATTTGATGCC
AGTTGGGAAAGAGACTGTTAAATATGAAGAGGAGCTTGATTTGCATGATGAAGAAGAGACCAGTGTTCCAGGAAGAC
CAGGTTCCACGAAACGAAGGCAGTGCTACCCAAAAGCAATTCCAGAGTGTTTGAGGGATAGTTATCCCAGACCTGAT
CAGCCCTGTTACCTGTATGTGATAGGAATGGTTTTAACTACACCTTTACCTGATGAACTCAACTTTAGAAGGCGGAA
GCTCTATCCTCCTGAAGATACCACAAGATGCTTTGGAATACTGACGGCCAAACCCATACCTCAGATTCCACACTTTC
CTGTGTACACACGCTCTGGAGAGGTTACCATATCCATTGAGTTGAAGAAGTCTGGTTTCATGTTGTCTCTACAAATG
CTTGAGTTGATTACAAGACTTCACCAGTATATATTCTCACATATTCTTCGGCTTGAAAAACCTGCACTAGAATTTAA
ACCTACAGACGCTGATTCAGCATACTGTGTTCTACCTCTTAATGTTGTTAATGACTCCAGCACTTTGGATATTGACT
TTAAATTCATGGAAGATATTGAGAAGTCTGAAGCTCGCATAGGCATTCCCAGTACAAAGTATACAAAAGAAACACCC
TTTGTTTTTAAATTAGAAGATTACCAAGATGCCGTTATCATTCCAAGATATCGCAATTTTGATCAGCCTCATCGATT
TTATGTAGCTGATGTGTACACTGATCTTACCCCACTCAGTAAATTTCCTTCCCCTGAGTATGAAACTTTTGCAGAAT
ATTATAAAACAAAGTACAACCTTGACCTAACCAATCTCAACCAGCCACTGCTGGATGTGGACCACACATCTTCAAGA
CTTAATCTTTTGACACCTCGACATTTGAATCAGAAGGGGAAAGCGCTTCCTTTAAGCAGTGCTGAGAAGAGGAAAGC
CAAATGGGAAAGTCTGCAGAATAAACAGATACTGGTTCCAGAACTCTGTGCTATACATCCAATTCCAGCATCACTGT
GGAGAAAAGCTGTTTGTCTCCCCAGCATACTTTATCGCCTTCACTGCCTTTTGACTGCAGAGGAGCTAAGAGCCCAG
ACTGCCAGCGATGCTGGCGTGGGAGTCAGATCACTTCCTGCGGATTTTAGATACCCTAACTTAGACTTCGGGTGGAA
AAAATCTATTGACAGCAAATCTTTCATCTCAATTTCTAACTCCTCTTCAGCTGAAAATGATAATTACTGTAAGCACA
GCACAATTGTCCCTGAAAATGCTGCACATCAAGGTGCTAATAGAACCTCCTCTCTAGAAAATCATGACCAAATGTCT
GTGAACTGCAGAACGTTGCTCAGCGAGTCCCCTGGTAAGCTCCACGTTGAAGTTTCAGCAGATCTTACAGCAATTAA
TGGTCTTTCTTACAATCAAAATCTCGCCAATGGCAGTTATGATTTAGCTAACAGAGACTTTTGCCAAGGAAATCAGC
TAAATTACTACAAGCAGGAAATACCCGTGCAACCAACTACCTCATATTCCATTCAGAATTTATACAGTTACGAGAAC
CAGCCCCAGCCCAGCGATGAATGTACTCTCCTGAGTAATAAATACCTTGATGGAAATGCTAACAAATCTACCTCAGA
TGGAAGTCCTGTGATGGCCGTAATGCCTGGTACGACAGACACTATTCAAGTGCTCAAGGGCAGGATGGATTCTGAGC
AGAGCCCTTCTATTGGGTACTCCTCAAGGACTCTTGGCCCCAATCCTGGACTTATTCTTCAGGCTTTGACTCTGTCA
AACGCTAGTGATGGATTTAACCTGGAGCGGCTTGAAATGCTTGGCGACTCCTTTTTAAAGCATGCCATCACCACATA
TCTATTTTGCACTTACCCTGATGCGCATGAGGGCCGCCTTTCATATATGAGAAGCAAAAAGGTCAGCAACTGTAATC
TGTATCGCCTTGGAAAAAAGAAGGGACTACCCAGCCGCATGGTGGTGTCAATATTTGATCCCCCTGTGAATTGGCTT
CCTCCTGGTTATGTAGTAAATCAAGACAAAAGCAACACAGATAAATGGGAAAAAGATGAAATGACAAAAGACTGCAT
GCTGGCGAATGGCAAACTGGATGAGGATTACGAGGAGGAGGATGAGGAGGAGGAGAGCCTGATGTGGAGGGCTCCGA
AGGAAGAGGCTGACTATGAAGATGATTTCCTGGAGTATGATCAGGAACATATCAGATTTATAGATAATATGTTAATG
GGGTCAGGAGCTTTTGTAAAGAAAATCTCTCTTTCTCCTTTTTCAACCACTGATTCTGCATATGAATGGAAAATGCC
CAAAAAATCCTCCTTAGGTAGTATGCCATTTTCATCAGATTTTGAGGATTTTGACTACAGCTCTTGGGATGCAATGT
GCTATCTGGATCCTAGCAAAGCTGTTGAAGAAGATGACTTTGTGGTGGGGTTCTGGAATCCATCAGAAGAAAACTGT
GGTGTTGACACGGGAAAGCAGTCCATTTCTTACGACTTGCACACTGAGCAGTGTATTGCTGACAAAAGCATAGCGGA
CTGTGTGGAAGCCCTGCTGGGCTGCTATTTAACCAGCTGTGGGGAGAGGGCTGCTCAGCTTTTCCTCTGTTCACTGG
GGCTGAAGGTGCTCCCGGTAATTAAAAGGACTGATCGGGAAAAGGCCCTGTGCCCTACTCGGGAGAATTTCAACAGC
CAACAAAAGAACCTTTCAGTGAGCTGTGCTGCTGCTTCTGTGGCCAGTTCACGCTCTTCTGTATTGAAAGACTCGGA
ATATGGTTGTTTGAAGATTCCACCAAGATGTATGTTTGATCATCCAGATGCAGATAAAACACTGAATCACCTTATAT
CGGGGTTTGAAAATTTTGAAAAGAAAATCAACTACAGATTCAAGAATAAGGCTTACCTTCTCCAGGCTTTTACACAT
GCCTCCTACCACTACAATACTATCACTGATTGTTACCAGCGCTTAGAATTCCTGGGAGATGCGATTTTGGACTACCT
CATAACCAAGCACCTTTATGAAGACCCGCGGCAGCACTCCCCGGGGGTCCTGACAGACCTGCGGTCTGCCCTGGTCA
ACAACACCATCTTTGCATCGCTGGCTGTAAAGTACGACTACCACAAGTACTTCAAAGCTGTCTCTCCTGAGCTCTTC
CATGTCATTGATGACTTTGTGCAGTTTCAGCTTGAGAAGAATGAAATGCAAGGAATGGATTCTGAGCTTAGGAGATC
TGAGGAGGATGAAGAGAAAGAAGAGGATATTGAAGTTCCAAAGGCCATGGGGGATATTTTTGAGTCGCTTGCTGGTG
CCATTTACATGGATAGTGGGATGTCACTGGAGACAGTCTGGCAGGTGTACTATCCCATGATGCGGCCACTAATAGAA
AAGTTTTCTGCAAATGTACCCCGTTCCCCTGTGCGAGAATTGCTTGAAATGGAACCAGAAACTGCCAAATTTAGCCC
GGCTGAGAGAACTTACGACGGGAAGGTCAGAGTCACTGTGGAAGTAGTAGGAAAGGGGAAATTTAAAGGTGTTGGTC
GAAGTTACAGGATTGCCAAATCTGCAGCAGCAAGAAGAGCCCTCCGAAGCCTCAAAGCTAATCAACCTCAGGTTCCC
AATAGCGGAAGCGCTGGTAGTGCTGGAAGTGGTGAACCCAATAGCGCAGGTGCCATGGTGAGCAAGGGCGAGGAGCT
GTTCACCGGGGTGGTGCCCATCCTGGTCGAGCTGGACGGCGACGTAAACGGCCACAAGTTCAGCGTGTCCGGCGAGG
GCGAGGGCGATGCCACCTACGGCAAGCTGACCCTGAAGTTCATCTGCACCACCGGCAAGCTGCCCGTGCCCTGGCCC

ACCCTCGTGACCACCCTGACCTACGGCGTGCAGTGCTTCAGCCGCTACCCCGACCACATGAAGCAGCACGACTTCTT
CAAGTCCGCCATGCCCGAAGGCTACGTCCAGGAGCGCACCATCTTCTTCAAGGACGACGGCAACTACAAGACCCGCG
CCGAGGTGAAGTTCGAGGGCGACACCCTGGTGAACCGCATCGAGCTGAAGGGCATCGACTTCAAGGAGGACGGCAAC
ATCCTGGGGCACAAGCTGGAGTACAACTACAACAGCCACAACGTCTATATCATGGCCGACAAGCAGAAGAACGGCAT
CAAGGTGAACTTCAAGATCCGCCACAACATCGAGGACGGCAGCGTGCAGCTCGCCGACCACTACCAGCAGAACACCC
CCATCGGCGACGGCCCCGTGCTGCTGCCCGACAACCACTACCTGAGCACCCAGTCCGCCCTGAGCAAAGACCCCAAC
GAGAAGCGCGATCACATGGTCCTGCTGGAGTTCGTGACCGCCGCCGGGATCACTCTCGGCATGGACGAGCTGTACAA
GTAATAAACCCGCTGATCAGCCTCGACTGTGCCTTCTAGTTGCCAGCCATCTGTTGTTTGCCCCTCCCCCGTGCCTT
CCTTGACCCTGGAAGGTGCCACTCCCACTGTCCTTTCCTAATAAAATGAGGAAATTGCATCGCATTGTCTGAGTAGG
TGTCATTCTATTCTGGGGGGTGGGGTGGGGCAGGACAGCAAGGGGGAGGATTGGGAAGACAATAGCAGGCATGCTGG
GGATGCGGTGGGCTCTATGGCTTCTGAGGCGGAAAGAACCAGCTGGGGCTCTAGGGGGTATCCCCACGCGCCCTGTA
GCGGCGCATTAAGCGCGGCGGGTGTGGTGGTTACGCGCAGCGTGACCGCTACACTTGCCAGCGCCCTAGCGCCCGCT
CCTTTCGCTTTCTTCCCTTCCTTTCTCGCCACGTTCGCCGGCTTTCCCCGTCAAGCTCTAAATCGGGGGCTCCCTTT
AGGGTTCCGATTTAGTGCTTTACGGCACCTCGACCCCAAAAAACTTGATTAGGGTGATGGTTCACGTAGTGGGCCAT
CGCCCTGATAGACGGTTTTTCGCCCTTTGACGTTGGAGTCCACGTTCTTTAATAGTGGACTCTTGTTCCAAACTGGA
ACAACACTCAACCCTATCTCGGTCTATTCTTTTGATTTATAAGGGATTTTGCCGATTTCGGCCTATTGGTTAAAAAA
TGAGCTGATTTAACAAAAATTTAACGCGAATTAATTCTGTGGAATGTGTGTCAGTTAGGGTGTGGAAAGTCCCCAGG
CTCCCCAGCAGGCAGAAGTATGCAAAGCATGCATCTCAATTAGTCAGCAACCAGGTGTGGAAAGTCCCCAGGCTCCC
CAGCAGGCAGAAGTATGCAAAGCATGCATCTCAATTAGTCAGCAACCATAGTCCCGCCCCTAACTCCGCCCATCCCG
CCCCTAACTCCGCCCAGTTCCGCCCATTCTCCGCCCCATGGCTGACTAATTTTTTTTATTTATGCAGAGGCCGAGGC
CGCCTCTGCCTCTGAGCTATTCCAGAAGTAGTGAGGAGGCTTTTTTGGAGGCCTAGGCTTTTGCAAAAAGCTCCCGG
GAGCTTGTATATCCATTTTCGGATCTGATCAGCACGTGATGAAAAAGCCTGAACTCACCGCGACGTCTGTCGAGAAG
TTTCTGATCGAAAAGTTCGACAGCGTCTCCGACCTGATGCAGCTCTCGGAGGGCGAAGAATCTCGTGCTTTCAGCTT
CGATGTAGGAGGGCGTGGATATGTCCTGCGGGTAAATAGCTGCGCCGATGGTTTCTACAAAGATCGTTATGTTTATC
GGCACTTTGCATCGGCCGCGCTCCCGATTCCGGAAGTGCTTGACATTGGGGAATTCAGCGAGAGCCTGACCTATTGC
ATCTCCCGCCGTGCACAGGGTGTCACGTTGCAAGACCTGCCTGAAACCGAACTGCCCGCTGTTCTGCAGCCGGTCGC
GGAGGCCATGGATGCGATCGCTGCGGCCGATCTTAGCCAGACGAGCGGGTTCGGCCCATTCGGACCGCAAGGAATCG
GTCAATACACTACATGGCGTGATTTCATATGCGCGATTGCTGATCCCCATGTGTATCACTGGCAAACTGTGATGGAC
GACACCGTCAGTGCGTCCGTCGCGCAGGCTCTCGATGAGCTGATGCTTTGGGCCGAGGACTGCCCCGAAGTCCGGCA
CCTCGTGCACGCGGATTTCGGCTCCAACAATGTCCTGACGGACAATGGCCGCATAACAGCGGTCATTGACTGGAGCG
AGGCGATGTTCGGGGATTCCCAATACGAGGTCGCCAACATCTTCTTCTGGAGGCCGTGGTTGGCTTGTATGGAGCAG
CAGACGCGCTACTTCGAGCGGAGGCATCCGGAGCTTGCAGGATCGCCGCGGCTCCGGGCGTATATGCTCCGCATTGG
TCTTGACCAACTCTATCAGAGCTTGGTTGACGGCAATTTCGATGATGCAGCTTGGGCGCAGGGTCGATGCGACGCAA
TCGTCCGATCCGGAGCCGGGACTGTCGGGCGTACACAAATCGCCCGCAGAAGCGCGGCCGTCTGGACCGATGGCTGT
GTAGAAGTACTCGCCGATAGTGGAAACCGACGCCCCAGCACTCGTCCGAGGGCAAAGGAATAGCACGTGCTACGAGA
TTTCGATTCCACCGCCGCCTTCTATGAAAGGTTGGGCTTCGGAATCGTTTTCCGGGACGCCGGCTGGATGATCCTCC
AGCGCGGGGATCTCATGCTGGAGTTCTTCGCCCACCCCAACTTGTTTATTGCAGCTTATAATGGTTACAAATAAAGC
AATAGCATCACAAATTTCACAAATAAAGCATTTTTTTCACTGCATTCTAGTTGTGGTTTGTCCAAACTCATCAATGT
ATCTTATCATGTCTGTATACCGTCGACCTCTAGCTAGAGCTTGGCGTAATCATGGTCATAGCTGTTTCCTGTGTGAA
ATTGTTATCCGCTCACAATTCCACACAACATACGAGCCGGAAGCATAAAGTGTAAAGCCTGGGGTGCCTAATGAGTG
AGCTAACTCACATTAATTGCGTTGCGCTCACTGCCCGCTTTCCAGTCGGGAAACCTGTCGTGCCAGCTGCATTAATG
AATCGGCCAACGCGCGGGGAGAGGCGGTTTGCGTATTGGGCGCTCTTCCGCTTCCTCGCTCACTGACTCGCTGCGCT
CGGTCGTTCGGCTGCGGCGAGCGGTATCAGCTCACTCAAAGGCGGTAATACGGTTATCCACAGAATCAGGGGATAAC
GCAGGAAAGAACATGTGAGCAAAAGGCCAGCAAAAGGCCAGGAACCGTAAAAAGGCCGCGTTGCTGGCGTTTTTCCA
TAGGCTCCGCCCCCCTGACGAGCATCACAAAAATCGACGCTCAAGTCAGAGGTGGCGAAACCCGACAGGACTATAAA
GATACCAGGCGTTTCCCCCTGGAAGCTCCCTCGTGCGCTCTCCTGTTCCGACCCTGCCGCTTACCGGATACCTGTCC
GCCTTTCTCCCTTCGGGAAGCGTGGCGCTTTCTCATAGCTCACGCTGTAGGTATCTCAGTTCGGTGTAGGTCGTTCG
CTCCAAGCTGGGCTGTGTGCACGAACCCCCCGTTCAGCCCGACCGCTGCGCCTTATCCGGTAACTATCGTCTTGAGT
CCAACCCGGTAAGACACGACTTATCGCCACTGGCAGCAGCCACTGGTAACAGGATTAGCAGAGCGAGGTATGTAGGC
GGTGCTACAGAGTTCTTGAAGTGGTGGCCTAACTACGGCTACACTAGAAGAACAGTATTTGGTATCTGCGCTCTGCT
GAAGCCAGTTACCTTCGGAAAAAGAGTTGGTAGCTCTTGATCCGGCAAACAAACCACCGCTGGTAGCGGTGGTTTTT
TTGTTTGCAAGCAGCAGATTACGCGCAGAAAAAAAGGATCTCAAGAAGATCCTTTGATCTTTTCTACGGGGTCTGAC

GCTCAGTGGAACGAAAACTCACGTTAAGGGATTTTGGTCATGAGATTATCAAAAAGGATCTTCACCTAGATCCTTTT
AAATTAAAAATGAAGTTTTAAATCAATCTAAAGTATATATGAGTAAACTTGGTCTGACAG
